# Diagnostic value of serum soluble triggering expressed receptor on myeloid cells 1 (sTREM-1) in suspected sepsis: a meta-analysis

**DOI:** 10.1186/s12865-020-0332-x

**Published:** 2020-01-13

**Authors:** Wei Chang, Fei Peng, Shan-Shan Meng, Jing-Yuan Xu, Yi Yang

**Affiliations:** Department of Critical Care Medicine, Zhongda Hospital, School of Medicine, Southeast University, Nanjing, China

**Keywords:** Sepsis, Systemic inflammatory response syndrome, SIRS, Diagnosis, Soluble triggering receptor expressed on myeloid cells 1, sTREM-1

## Abstract

**Background:**

We aim to synthesize the up-to-date studies to investigate the diagnostic value of serum soluble triggering expressed receptor on myeloid cells 1 (sTREM-1) in suspected sepsis.

**Results:**

A total of 19 studies with 2418 patients were finally enrolled in the meta-analysis. The pooled sensitivity was 0.82 (95% CI 0.73 to 0.89), specificity 0.81 (95% CI 0.75 to 0.86), positive likelihood ratio 4.3 (95% CI 3.02 to 6.12), negative likelihood ratio 0.22 (95% CI 0.24 to 0.35), diagnostic odds ratio 20 (95% CI 9 to 41) and AuROC 0.88 (95% CI 0.85 to 0.91). The meta-regression analysis revealed that the sample size, reference standard description, prevalence of sepsis in the trials and consecution of patient recruitment might be the source of heterogeneity.

**Conclusions:**

The serum sTREM-1 had a moderate ability in diagnosis in suspected sepsis based on the current studies. However, more large-scale studies were needed to further evaluate the diagnostic accuracy of sTREM-1.

## Background

Sepsis is defined as life-threatening organ dysfunction caused by a dysregulated host response to infections, which causes high mortality in the intensive care unit (ICU) and is a grave burden to the public health [1] . Early recognition and diagnosis of sepsis in the high-risk patients with suspected infection is essential for the prompt management and empirical antibiotics therapy, which could potentially improve the mortality in septic patients [2].

The utility of biomarkers in the early recognition, risk stratification, antibiotic stewardship and outcome prediction in septic patients has long been applied in the clinical practice [3]. A myriad of molecules has been under investigation in the early discrimination of sepsis, including C-reactive protein, procalcitonin, cytokines and surface markers of circulating leukocytes [4], which could be promising biomarkers in the diagnosis of sepsis.

Triggering receptor expressed on myeloid cells 1 (TREM-1), a member of immunoglobulin family predominantly expressed on the neutrophils and monocytes, was first identified by Bouchon A. et al in 2000 [5], which is upregulated in response of bacterial and fungal infections but poorly expressed in non-infectious inflammation [6, 7] . The soluble form of TREM-1 (sTREM-1) is shed from cell surface and released into body fluids including plasma, pleural effusion, sputum and urine during the process of infections through proteolytic cleavage by metalloproteinases triggered by lipopolysaccharide [8, 9].

The elevated sTREM-1 in the body fluids during infection could be measured directly by immunosorbent assays and used as a tool in discriminating infection from non-infectious inflammation, which makes it a promising candidate in the diagnosis of sepsis. A plethora of studies have been conducted to investigate the value of sTREM-1 as an early biomarker in patients with suspected infections since its discovery [10], however, its diagnostic accuracy remains undetermined.

Previous meta-analyses has evaluate the diagnostic ability of sTREM-1 in suspected infections [11–13], however with the burgeoning clinical studies of sTREM-1 as a diagnostic toolkit in suspected sepsis in recent years, we thought it quite necessary to conduct an up-to-date meta-analysis to assess the diagnostic value of serum sTREM-1 in patients with suspected sepsis.

## Results

### Study selection

**Fig. 1:**
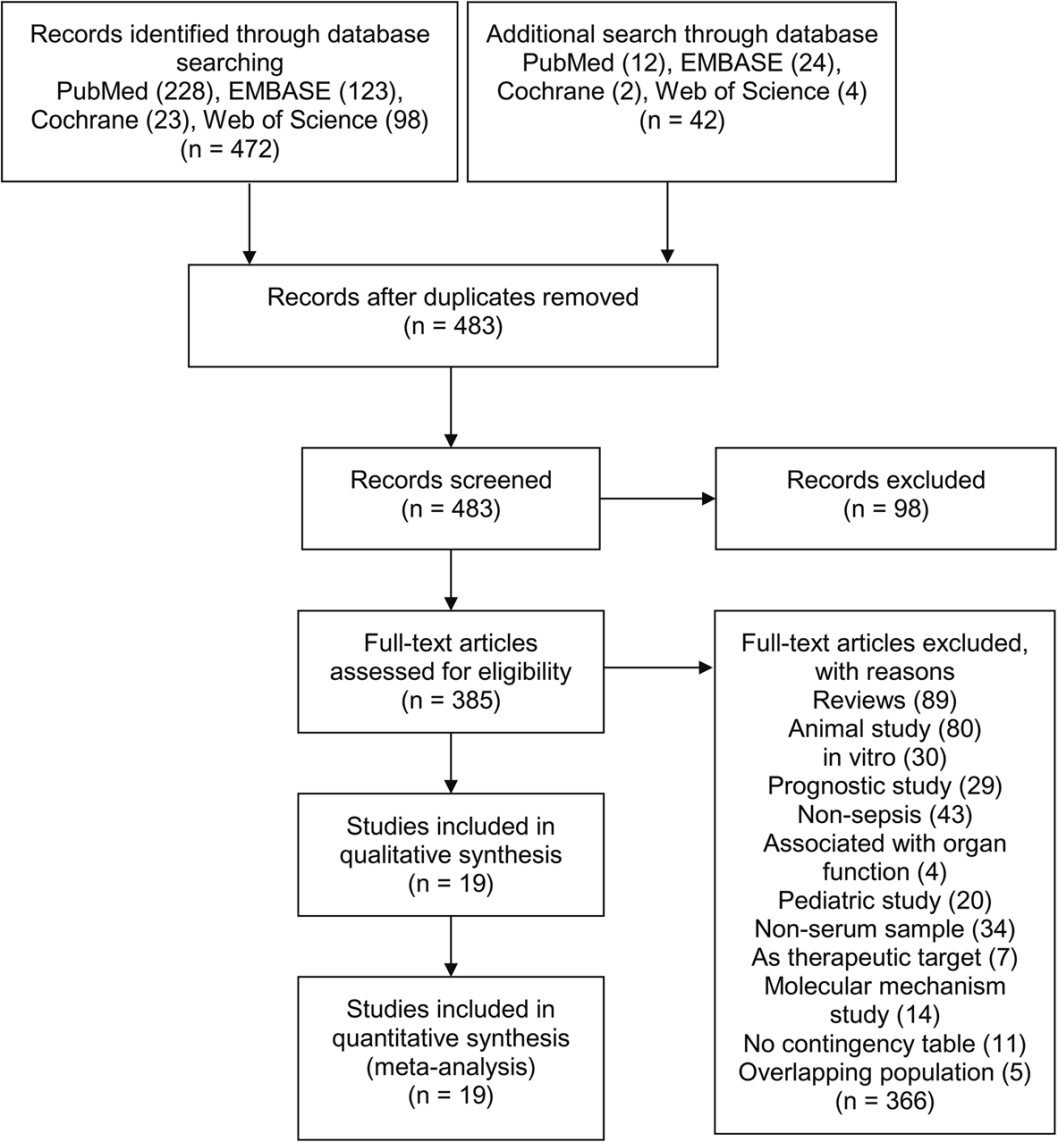
Flow diagram. Flow chart of study screen and selection

A total of 514 abstracts were recruited from the search, 31 duplicates were excluded and the remaining 483 were left for screen, within which 98 abstracts were not eligible. In the remaining 385 abstracts, full manuscripts were recruited for further assessment, and 366 articles were excluded with reasons. A final of 19 studies were included in this meta-analysis [14–32] (Fig. 1).

### Study characteristics

**Fig. 2:**
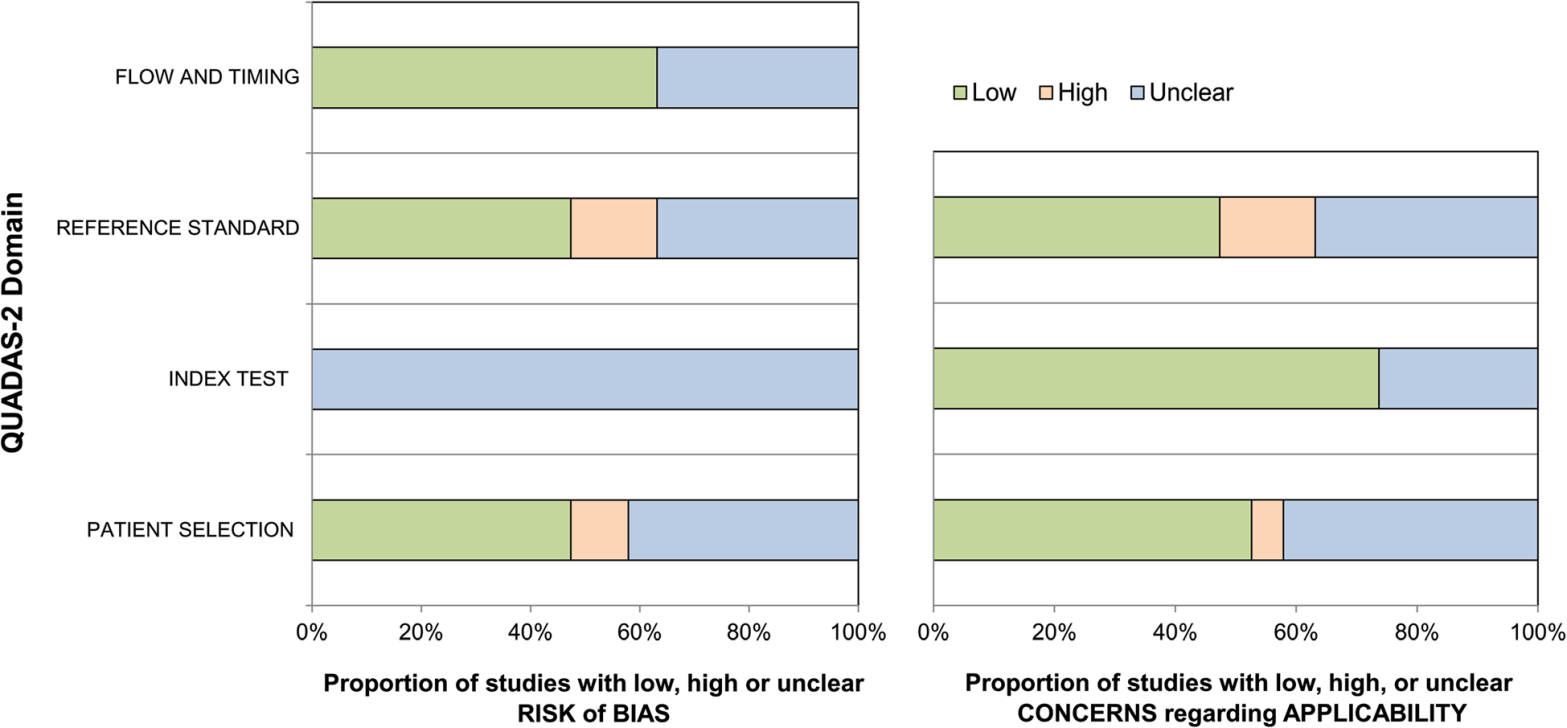
The qualities of the included studies assessed by QUADAS-2 tool

A total of 2418 patients was finally enrolled in this meta-analysis, with the average sepsis prevalence of 60.3%. Among the 19 studies included, 17 were prospective [14, 15, 17, 19–32] and two were cross-sectional [16, 18]; in five studies traumatic or post-operative patients were enrolled exclusively [16, 19, 22, 28, 29]. Pulmonary infection was the leading cause of infection in 14 studies. Two studies focused on pulmonary [32] or abdominal infections [28] exclusively. The cut-off values ranged from 30 pg/mL to 60 ng/mL, sensitivity from 49 to 98.3%, specificity from 40 to 91.7% and AuROC from 0.61 to 0.978. Three articles were written in Chinese [17, 26, 32]. (Table 1) The quality assessments of the included studies (Additional file 3) were summarized in Fig. 2.

**Table 1:** Characteristics of the included studies. ICU intensive care unit, SIRS systemic inflammatory response syndrome, TP true positive, FP false positive, TN true negative, FN false negative, ED emergency department, AuROC area under curve, HAP hospital-acquired pneumonia, NR not reported. * within which 15 patients did not have sample available for analysis, † A total of 117 patients were determined with infection, within which 96 patients were bacterial infection, 16 patients were viral infection and five patients were parasite infection. Soluble TREM-1 was used to diagnose bacterial infections in SIRS

Study	Country	Setting	Study Design	Patients	Infection Characteristics	Test Timing	Assay Method	Sepsis Prevalence/Mortality (%)	Cut-off (pg/mL)	Sensitivity/Specificity(%)	TP	FP	FN	TN	AuROC
Aksaray S et al., 2016 [14]	Turkey	Medical-surgical ICU	Prospective consecutive	90 patients with SIRS	Lung (44.2%) and blood (21%)	Within 24 h of admission	ELISA (MyBioSource, Inc., San Diego, CA, USA)	57.8 (52)/32.7 (17)	133	71.15/76.32	37	9	15	29	0.78
Barati M et al., 2010 [15]	Iran	Medical and surgical ICU	Prospective consecutive	95 patients with SIRS, 37 non-SIRS patients as control	Not reported	Upon admission at ICU	Quantitative sandwich enzyme immunoassay (Quantikine, R&D Systems, Inc., Minneapolis, USA)	54.7 (52 in 95)/NR	725	70/60	36	17	16	26	0.65
Brenner T et al., 2016 [16]	Germany	Surgical ICU and post-operative care	Re-analysis of prospective cohort	60 patients with septic shock, 30 post-operative control and 30 healthy volunteers	GI tract (53.3%), others (30%) and lung (20%); Gram-positive (26.7%), Gram-negative (26.7%)	At sepsis onset, 24 h, 4 days, 7 days, 14 days and 28 days	ELISA (R&D Systems, Inc., Minneapolis, MN, USA)	66.7 (60 in 90)/NR	30	98.3/90	59	3	1	27	0.955
Dong Y et al., 2012 [17]	China	Emergency and medical ICU	Prospective	64 patients with SIRS	Respiratory (60.5%), abdominal (14%) and biliary tract (5%)	Within 24 h of recruitment, day 4 and 7	ELISA (R&D Systems, Inc., Minneapolis, MN, USA)	67.2 (43)/32.5 (14)	95.9	76.7/90.5	33	2	10	19	0.868
Gamez-Diaz LY et al., 2011 [18]	Colombia	ED	Cross-sectional study with prospective data	631 patients with possible sepsis syndrome*	CAP (22%), urinary tract (16%) and soft tissue (16%)	Within 24 h of the first ED evaluation	ELISA (Quantikine, R&D Systems, Inc., Minneapolis, MN, USA)	65.7 (405 in 616)/13.5 (56)	134	60/59.2	243	86	162	125	0.614
Giamarellos-Bourboulis EJ et al., 2008 [19]	Greece	ICU	Prospective	69 severely injured patients (ISS > 25) with SIRS, 10 patients with ISS > 25 without SIRS as control group	HAP (79%), acute pyelonephritis (7%) or primary gram-negative bacteremia (14%)	At admission, day 4, 7 and 15; and within 24 h after the diagnosis of any septic complications	homemade enzyme immunosorbent assay	62.3 (43 in 69)/34.9 (15)	40	56.5/91.7	24	2	19	24	0.708
Gibot S et al., 2004 [20]	France	Medical ICU	Prospective consecutive	76 patients with SIRS	Respiratory tract (55%), abdominal (22%) and genitourinary tract (11%); 55% gram-negative and 42% gram-positive in 40 microbiological proven patients	Within 12 h after admission	immunoblots	61.8 (47)/32 (15)	60,000	96/89	45	3	2	26	0.97
Gibot S et al., 2012 [21]	France	ICU	Prospective consecutive	300 patients with SIRS	Lung (49.4%), abdomen (12.3%) and Genitourinary (11%); positive microbiological documents in 88 (57%) pats, with 55% gram-positive and 45% gram-negative	Within 12 h after admission	ELISA (Quantikine, R&D Systems, Inc., Minneapolis, MN, USA)	51.3 (154)/26 (40)	755	53.2/86.3	82	20	72	126	0.73
Halim B et al., 2015 [22]	Turkey	Hospitalized patients	Prospective	74 patients with SIRS	Respiratory tract (39.4%), GI tract (24.2%) and urinary tract (21%); Gram-positive (21.2%), Gram-negative (60.6%)	On day 0 at admission	ELISA (R&D Systems, Inc., Minneapolis, MN, USA)	44.6 (33)/54.5 (18)	199.72	81.8/73.2	27	11	6	30	0.826
Kofoed K et al., 2007 [23]	Denmark	Department of infectious disease and medical ED	Prospective consecutive	151 patients with SIRS	Respiratory (60.4%), urinary tract (26%) and GI tract (17%)	At inclusion	Luminex multiplex assay (Luminex corp. Austin, TX, USA)	63.6 (96 in 151†)/NR	3500	82/40	79	33	17	22	0.61
Latour-Perez J et al., 2010 [24]	Spain	General ICU	Prospective	114 patients with SIRS	Respiratory (40%), abdominal-pelvis (21%) and urinary (12.5%)	As soon as the detection of SIRS	ELISA (R&D Systems, Inc., Minneapolis, MN, USA)	63.2 (72)/37.5 (27)	463.2	49/79	35	9	37	33	0.62
Li L et al., 2013 [25]	China	Surgical ICU	Prospective consecutive	52 post-operative patients with SIRS	60.5% infected with bacteria, 5.3% with fungi, 28.9% both bacteria and fungi; among 34 patients infected w bacteria, 14 with bacillus, 20 with cocci	Within 12 h after admission	ELISA (R&D Systems, Inc., Minneapolis, MN, USA)	73.1 (38)/48 (25)	73.57	79/79	30	3	8	11	0.82
Li Z et al., 2016 [26]	China	ICU	Prospective consecutive	80 patients with SIRS, 25 healthy volunteers	Respiratory (48%), urinary tract (22%) and abdominal (14%)	First day at admission	ELISA (R&D Systems, Inc., Minneapolis, MN, USA)	62.5 (50 in 80)/30 (15)	123.5	76/76.6	38	7	12	23	0.862
Rivera-Chavez FA et al., 2009 [27]	USA	Surgical ICU	Prospective	93 patients with SIRS, 15 patients with ISS > 25 without SIRS as control group	Lung (60%), abdomen (13%) and blood (12%); 28 (30%) patients with gram-negative isolation, 22 (23%) with gram-positive isolation, and 6 (7%) with fungus	Within 24-36 h after admission	DuoSet enzyme-linked immunosorbent assay (R&D Systems, Inc., Minneapolis, MN, USA)	60.2 (56 in 93)/11 (6)	230	98/91	55	4	1	33	0.97
Song X et al., 2017 [28]	China	Department of gastrointestinal surgery	Prospective	128 SIRS patients after abdominal operation, and 60 healthy controls	Intestinal fistula (23.5%), gastric fistula (19.1%) and ileus (22.1%)	Within 24 h after hospitalization	ELISA (Quantikine, R&D Systems, Inc., Minneapolis, MN, USA)	53.1 (68 in 128)/21.4 (12)	113.06	80/76	54	14	14	46	0.82
Soud DEM et al., 2011 [29]	Egypt	Surgical ER and ICU of anesthesia	Prospective	70 trauma patients with SIRS, 10 non-SIRS trauma patients as control group	Abdomen (31.6%), chest (26.3) and urinary (15.8%)	Not reported	ELISA (Quantikine, R&D Systems, Inc., Minneapolis, MN, USA)	27.1 (19 in 70)/NR	254	94.7/91.8	18	4	1	47	NR
Su L et al., 2013 [30]	China	Respiratory, Surgical and Emergency ICU	Prospective	130 patients with SIRS	Pulmonary (83%), post-operative (31%) and urinary tract (24%); Gram-positive (37%), Gram-negative (81%) and fungi (62%)	Within 24 h after admission, and in day 3, 5, 7, 10 and 14	ELISA (Quantikine, R&D Systems, Inc., Minneapolis, MN, USA)	76.9 (100 in 130)/43 (43)	64.4	91/89.6	91	3	9	27	0.978
Wang H et al., 2011 [31]	China	ICU	Prospective consecutive	56 patients with SIRS, 25 non-SIRS as control group	Not reported	Within 24 h after hospitalization	ELISA (Quantikine, R&D Systems, Inc., Minneapolis, MN, USA)	57.1 (32)/34 (11)	135	93.8/84.7	30	4	2	20	0.935
Yang J et al., 2014 [32]	China	ICU	Prospective	70 patients with SIRS, 30 non-SIRS as control group	Pneumonia	At day 1, 4 and 7 of admission in sepsis, at day 1 and 4 in SIRS	ELISA (Westang Bio-technology Co., Ltd., Shanghai, China)	55.7 (39 in 70)/38 (15)	172.15	78.9/82.1	31	6	8	25	0.796

### Syntheses of results

**Fig. 3:**
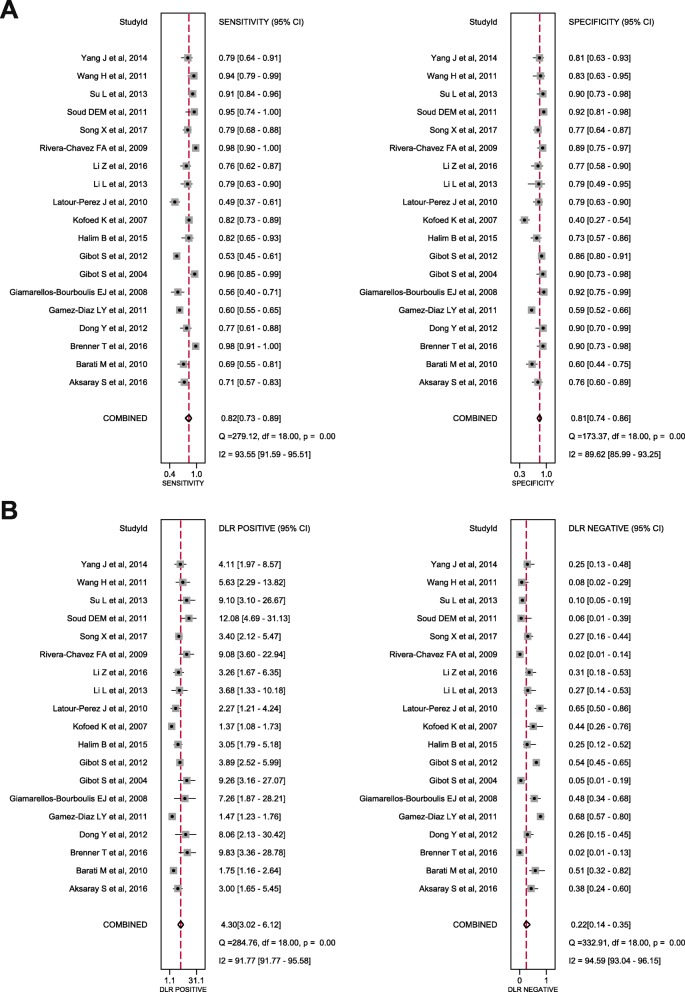
Forest plots. **a** Forest plots showing the sensitivity (0.82, 95% CI 0.73–0.89) and specificity (0.81, 95% CI 0.74–0.86) of sTREM-1 in diagnosis in suspected sepsis; **b** Forest plots showing the positive likelihood ratio (4.20, 95% CI 3.02–6.12) and negative likelihood ratio (0.22, 95% CI 0.14–0.35) of sTREM-1 in diagnosis in suspected sepsis

**Fig. 4:**
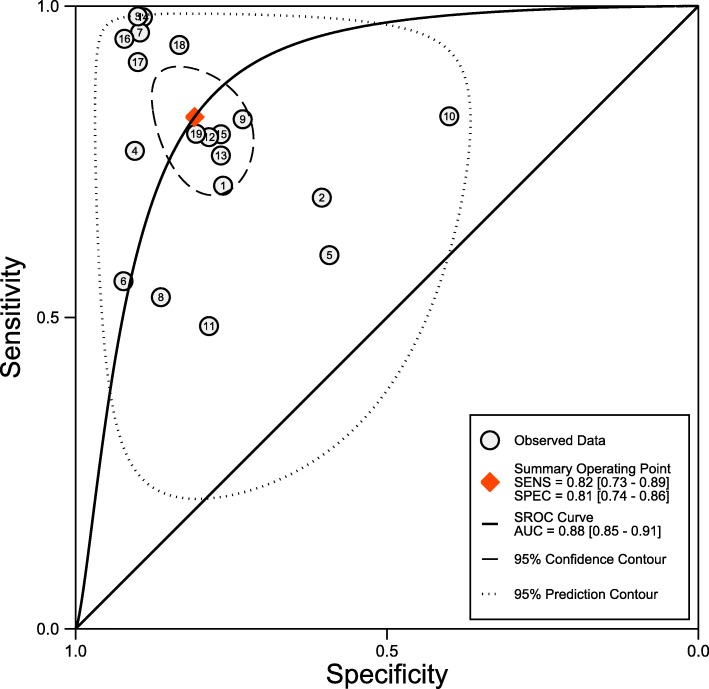
Summary receiver operating curve

**Fig. 5:**
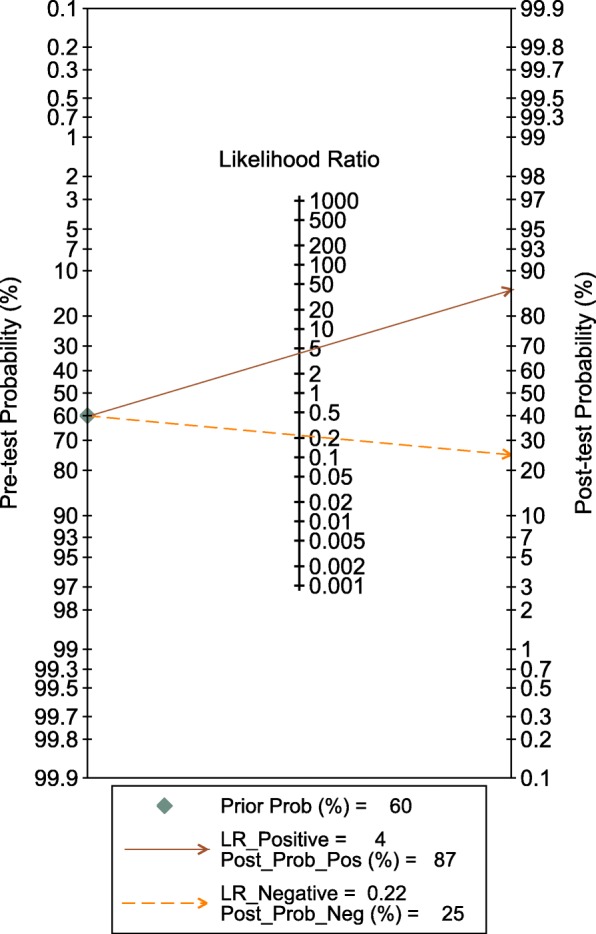
Fagan’s nomogram. Pre-test probability was set at 60%, which yielded a post-test positive probability of 87% and negative of 26%

**Fig. 6:**
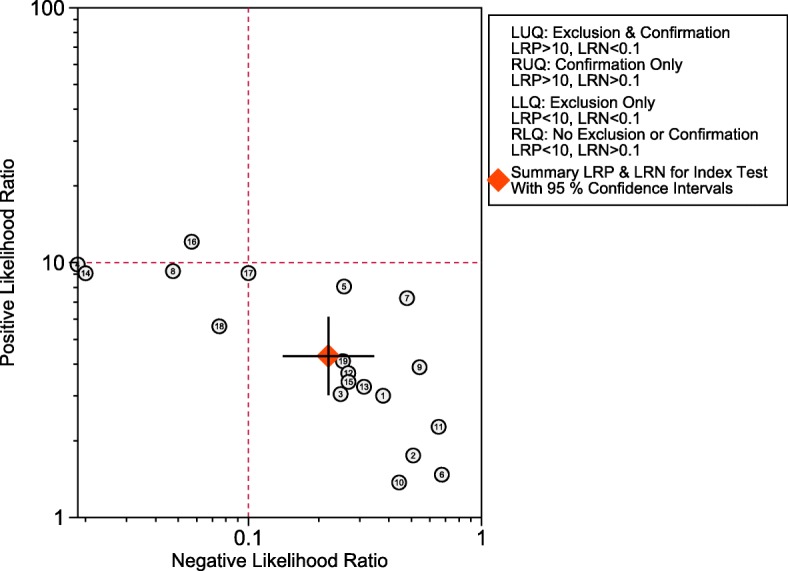
Scattergram. The overall diagnostic accuracy of sTREM-1 was at the right lower quadrant with PLR < 10 and NLR > 0.1, which implying no exclusion or confirmation

The synthesis of the 19 studies by the bivariate model yielded a pooled sensitivity of 0.82 (95% CI 0.73 to 0.89), specificity of 0.81 (95% CI 0.74 to 0.86), PLR of 4.3 (95% CI 3.0 to 6.1), NLR of 0.22 (95% CI 0.14 to 0.35) and DOR of 20 (95% CI 9 to 41) (Fig. 3), with AuROC of 0.88 (95% CI 0.85 to 0.91) (Fig. 4). The proportion of heterogeneity likely due to threshold effect was 0.20. We assumed the pre-test probability of 60% as the overall average sepsis prevalence concluded from the trials enrolled and yielded the post-test positive probability of 87% and negative of 26%, as illustrated in the Fagan’s nomogram (Fig. 5). The scattergram indicated that the sTREM-1 of limited clinical diagnostic value with PLR < 10 and NLR > 0.1 (no exclusion or confirmation) (Fig. 6).

### Risk of bias and sub-group analysis

The studies were divided according to the cut-off values of sTREM-1, and the results revealed the AuROC of 0.87 (95% CI 0.84 to 0.90), sensitivity of 0.81 (95% 0.73 to 0.87) and specificity of 0.80 (95% CI 0.73 to 0.86) at the cut-off range of 30 to 199.72 pg/mL; and AuROC of 0.89 (95% CI 0.86 to 0.91), sensitivity of 0.85 (95% CI 0.64 to 0.95) and specificity of 0.80 (95% CI 0.65 to 0.90) at the cut-off range of 230 pg/mL to 60 ng/mL (Additional file 4).

We conducted the meta-analysis in the sub-group of 7 prospective trials conducted in the ICU, in which the patients with SIRS were consecutively recruited [14, 15, 20, 21, 25, 26, 31]. In this relatively homogenous population of patients, the result revealed that the pooled sensitivity of 0.80 (95% CI 0.68 to 0.89), specificity of 0.76 (95% CI 0.64 to 0.84), PLR of 3.3 (95% CI 2.0 to 5.5), NLR of 0.26 (95% 0.14 to 0.48) and DOR of 13 (95% CI 4 to 38) (Additional file 5: Figure S1).

**Fig. 7:**
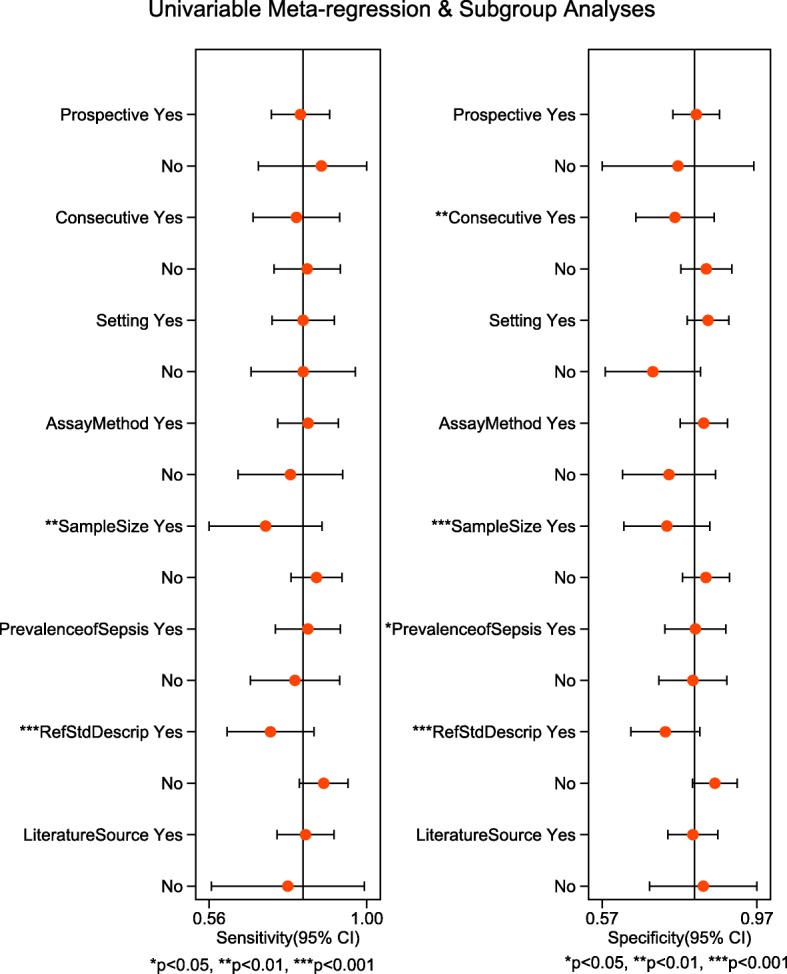
Univariate meta-regression. The following factors were included in the meta-regression: 1. Prospective (prospective – yes; cross-sectional – no); 2. Consecutive (consecutive enrollment – yes; otherwise – no); 3. Setting (in ICU – yes; otherwise – no); 4. AssayMethod (ELISA branded R&D – yes; otherwise – no); 5. SampleSize (sample size more than 100 – yes; sample size less than or equal to 100 – no); 6. PrevalenceofSepsis (sepsis prevalence > 60% – yes; sepsis prevalence <=60% – no); 7. RefStdDescrip (reference standard described – yes; no description of reference standard – no); 8. LiteratureSource (literature source in English – yes; non-English source – no)

A categorical univariate meta-regression analysis was conducted including the factors which could potentially bring bias to the results. We found that the sample size, and the reference standard description was possibly related with the heterogeneity in both sensitivity and specificity, and the prevalence of sepsis and the consecution of patient recruitment was possibly related with the heterogeneity in specificity (Fig. 7). Sub-group analyses were conducted according to the factors possibly related to the heterogeneity. (Additional file 6: Figure S2).

**Fig. 8:**
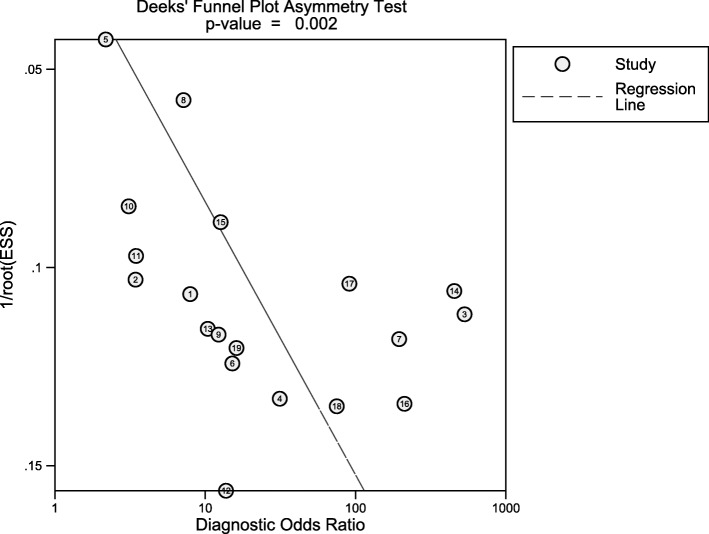
Deeks’ funnel plot. The *P* value for the slope coefficient indicating significant asymmetry was 0.02, which indicating a high likelihood of publication bias

The Deek’s funnel plot was constructed and potential bias could be inspected with the *P* value of 0.002, suggesting publication bias should be considered (Fig. 8).

## Disscussion

In this study, we synthesized 19 trials and found that the sTREM-1 has a moderately accuracy in diagnosis of sepsis in high-risk patients with the pooled sensitivity of sensitivity of 0.82 (95% CI 0.73 to 0.89), specificity of 0.81 (95% CI 0.74 to 0.86) and the AuROC of 0.88 (95% CI 0.85 to 0.91).

In previous meta-analysis by Wu et al including 11 trials of 1795 patients, they found the pooled sensitivity and specificity of 0.79 (95% CI 0.65 to 0.89) and 0.80 (95% CI 0.69 to 0.88) respectively, and DOR of 4.0 (95% CI 2.4 to 6.9) [12], and in our study with enlarged sample size enrolling 19 trials of 2418 patients saw improved accuracy in diagnosis in patients with suspected sepsis.

However, the sample size of the trials we enrolled in this study was relatively small (in most of studies the participants not exceeding 100), which could lead to type II error with elevated false positive [33]. We thus conducted the sub-group analysis including studies enrolling more than 100 patients and found the pooled sensitivity of 0.71 (95% CI 0.58 to 0.82), specificity of 0.69 (95% CI 0.54 to 0.81) and DOR of 5.48 (2.10 to 14.27), which showed that the sensitivity, specificity and DOR all dropped compared with the overall results, suggesting more large-scale trials are needed for the evaluation of the diagnostic ability of sTREM-1.

The baseline characteristics of patients was complicated (post-operative, traumatic, mechanical ventilated), and heterogeneity in the patient selection might bring bias to the results; the severity of sepsis was different between studies with mortality ranged from 11% [27] to 54.4% [22], suggesting different pathogen virulence, inflammatory response and organ dysfunction, could probably influence the serum sTREM-1 level and its ability in discrimination of sepsis from SIRS. In some of the studies we included, not only patients with bacterial or fungal, but also with viral or parasite infection were recruited [23], and the heterogeneous composition of infection may also potentially bring bias to the results. The diagnosis of sepsis was relied on the comprehensive combination of clinical manifestation, laboratory and radiographic results, and the microbiological isolation or even in some occasions, the response to the empirical antibiotics therapy. We noticed that in the studies we included, the authors used inconsistent methods of determination of infection and in some studies only patients with positive pathogen isolation were included in the sepsis group [22, 27], all of which could potentially cause biases.

The level of serum sTREM-1 was also reported to be elevated in some non-infectious diseases in recent years, including chronic kidney disease on hemodialysis [34], chronic obstructive pulmonary disease [35], inflammatory bowel disease [36], atherosclerosis [37] and in malignant carcinomas like hepatocellular carcinoma [38] and non-small cell lung cancer [39], which could affect the specificity of sTREM-1 in sepsis diagnosis.

An ideal biomarker for sepsis should give information to the syndrome recognition, precise diagnosis and prognosis, and improve antibiotic stewardship [3], but unfortunately, no single biomarker could accomplish this task currently. In our study, sTREM-1 yielded a moderate diagnostic accuracy of sepsis. Currently, combination of biomarker for the diagnosis and prognostication of sepsis have been widely investigated as in the trial by Gibot et al [21], serum sTREM-1 were combined with PMN CD64 index and procalcitonin in diagnosis of sepsis.

### Limitations

This study had several limitations. The sample size in most of the trials we included in this meta-analysis was relatively small (less than 100 participants). In some trials, the predictive ability of sTREM-1 in sepsis was also evaluated, however, was not included in our study. All the cut-off values in the trials included were not pre-specified, but optimized by the sensitivity and specificity, so the cut-off value of sTREM-1 for sepsis diagnosis was not determined so far.

## Conclusions

Serum sTREM-1 has a moderate accuracy in diagnosis in patients with suspected sepsis, however, the heterogeneity was high between studies. More large-scale studies are needed for validation the diagnostic value of sTREM-1 in suspected sepsis.

## Methods

This manuscript was prepared following the guidelines of Preferred Reporting Items for Systemic Reviews and Meta-analyses (PRISMA) statement [40, 41] and Meta-analysis of Observational Studies in Epidemiology (MOOSE) statement [42]. This study has been registered in PROSPERO (CRD42018083695).

### Eligibility criteria

We aimed to include all the clinical trials investigating serum sTREM-1 as an early biomarker in the patients with suspected sepsis. The including criteria were as follows: (1) clinical trials of adult patients (> 18 year-old) with suspected sepsis; (2) serum or plasma sTREM-1 protein expression was measured, if multiple measures were taken in the studies, only the earliest one was used; (3) a 2 × 2 contingency table with true positive (TP), false positive (FP), false negative (FN) and true negative (TN) could be constructed. As most of studies were conducted in prior of the upgrade to sepsis-3, sepsis was defined in accordance with the Surviving Sepsis Campaign Guidelines (2012) as the presence of infections together with the manifestation of systemic inflammatory response syndrome (SIRS) [43], and the determination of infection was remained to the researchers in individual studies.

### Information sources

Two reviewers searched the electronic database including PubMed, EMBASE, Cochrane Central Register and Web of Science updated to June, 2018 separately, with no language restrictions. When relevant reviews or meta-analysis were reviewed, a backwards snowballing search was conducted for further studies.

### Search

The following key words were used in our search strategy: “soluble triggering expressed receptor on myeloid cells 1”, “triggering expressed receptor on myeloid cells 1”, “sTREM-1”, “TREM-1”, “sepsis”, “severe sepsis”, “pyemia” and “septicemia”. (Additional file 1).

### Study selection

The titles and abstracts of the articles initially reviewed separately by two reviewers, and the full manuscripts were recruited if potentially relevant for further assessment. Disagreements were solved by consensus.

### Data extractions

The following information was extracted for the analysis and assessment of the potential bias: (1) characteristics of study (design, settings, inclusion and exclusion criteria) and participants (sepsis prevalence, infection sites and microbiological features); (2) assay methods, cut-offs, sensitivity, specificity and the area under ROC curve (AuROC); (3) the procedures of diagnosis (may including the clinical manifestation, laboratory and radiographic results, microbiological isolations); (4) the time point of sample obtain and diagnosis.

### Assessment of risk of Bias

The internal validity and risk of bias of the included studies were evaluated by the Quality Assessment of Diagnostic Accuracy Studies 2 (QUADAS-2) tool [44], which consist of four domains including patient selection, index test, reference standard, and flow and timing, with the risk of bias assessed as “low”, “unclear” and “high”. Discrepancies were solved by consensus. (Additional file 2).

### Statistical analysis

The number of patients classified in TP, FP, FN and TN were calculated from the prevalence of sepsis, sensitivity and specificity as provided in the studies. Data synthesis was performed within the bivariate mixed-effects regression framework to calculate average sensitivity and specificity, and also positive/negative likelihood ratio (PLR & NLR) and diagnostic odds ratio (DOR, defined as PLR divided by NLR, which reflected the effectiveness of diagnosis), presented with 95% confidence intervals (CI) [45].

The derived logit estimates of sensitivity, specificity and respective variance were used to construct a summary ROC curve, and the area under ROC (AuROC) was calculated for the global measure of the test performance, with 0.5 > = AuROC <= 0.7 as low, 0.7 > = AuROC <= 0.9 as moderate, and 0.9 > = AuROC <= 1 as high diagnostic accuracy [46]. The threshold effects were also visually assessed from the summary ROC, and the proportion of variance due to threshold effects was calculated as the squared correlation coefficient estimated from the between-study covariance parameter tested by rank correlation test.

Post-test probability was calculated using likelihood ratios based on Bayes’ theorem and depicted visually with Fagan’s nomograms [47]. The likelihood ratio scattergram was also plotted, with the definition of left upper quadrant of exclusion and confirmation, right upper quadrant of confirmation only, left lower quadrant of exclusion only and right lower quadrant of no exclusion or confirmation, respectively [48].

The factors that could potentially bring heterogeneity were extract and introduced in the univariate meta-regression analysis, as we speculated from the designation of the studies, including study design (prospective or not, and consecutive or not), settings (in the ICU exclusively or not), assays (methods and kit brands), sample size (> 100 participants or not), prevalence of sepsis (according to the average prevalence of sepsis from the studies included), the description of the reference standard (described in detail or not) and literature source (published in English or not). Sub-analyses were subsequently conducted according to the results of the univariate meta-regression to investigate the diagnostic performances of sTREM-1 in sub-group population of patients.

The Deeks’ funnel plot was used to evaluate the publication bias, with *P* value < 0.10 for the slope coefficient indicating significant asymmetry and a high likelihood of publication bias [49].

STATA (ver. 14, StataCorp LP, TX, USA) was used for the analyses, and *midas* command was used for the calculations. A two-tailed *P* value < 0.05 was considered statistical significance.

## Supplementary information

**Additional file 1.** Full electronic search strategy on PubMed.

**Additional file 2.** Data extraction and study quality assessment protocol.

**Additional file 3.** Detailed characteristics and quality assessment of the included studies.

**Additional file 4.** Sub-group analysis according to the cut-off values.

**Additional file 5: Figure S1.** Sub-group analysis of studies conducted in ICU with patients consecutively recruited**.** The sub-group of 7 prospective trials conducted in the ICU, in which the patients with SIRS were consecutively recruited. A. Forest plots showing the sensitivity (0.80, 95% CI 0.68–0.89) and specificity (0.76, 95% CI 0.64–0.8*4*) of sTREM-1; B. Forest plots showing the positive diagnostic likelihood ratio (DLR positive) (3.30, 95% CI 1.98–5.50) and negative diagnostic likelihood ratio (DLR negative) (0.26, 95% CI 0.14–0.48) of sTREM-1.

**Additional file 6: Figure S2.** Sub-group analyses according to the meta-analysis results.

## Data Availability

The datasets used and/or analysed during the current study available from the corresponding author on reasonable request.

## Abbreviations

AuROC: Area under ROC
CI: Confidence interval
DOR: Diagnostic odds ratio
FN: False negative
FP: False positive
ICU: Intensive care unit
MOOSE: Meta-analysis of observational studies in epidemiology
NLR: Negative likelihood ratio
PLR: Positive likelihood ratio
PRISMA: Preferred reporting items for systemic reviews and meta-analyses
QUADAS-2: Quality assessment of diagnostic accuracy studies 2
ROC: Receiver operating characteristics
SIRS: Systemic inflammatory response syndrome
sTREM-1: soluble triggering receptor expressed on myeloid cells 1
TN: True negative
TP: True positive
TREM-1: Triggering receptor expressed on myeloid cells 1
