# Supplementary material for: Diagnostic value of serum soluble triggering expressed receptor on myeloid cells 1 (sTREM-1) in suspected sepsis: a meta-analysis
Source: BMC Immunol. 2020 Jan 13;21:2. doi: 10.1186/s12865-020-0332-x (PMC6958609; doi:10.1186/s12865-020-0332-x)
Supplement: Supplementary file 1 — Additional file 1. Full electronic search strategy on PubMed. [file 12865_2020_332_MOESM1_ESM.pdf]

((((((soluble triggering expressed receptor on myeloid cells 1) OR triggering expressed receptor on myeloid cells 1) OR sTREM-1) OR TREM1) OR TREM-1)) AND ((((sepsis) OR severe sepsis) OR septicemia) OR pyemia)
