# Supplementary material for: Diagnostic value of serum soluble triggering expressed receptor on myeloid cells 1 (sTREM-1) in suspected sepsis: a meta-analysis
Source: BMC Immunol. 2020 Jan 13;21:2. doi: 10.1186/s12865-020-0332-x (PMC6958609; doi:10.1186/s12865-020-0332-x)
Supplement: Supplementary file 2 — Additional file 2. Data extraction and study quality assessment protocol. [file 12865_2020_332_MOESM2_ESM.pdf]

DATA EXTRACTION AND STUDY QUALITY ASSESSMENT PROTOCOL

The qualities of the articles would be assessed by the Quality Assessment of Diagnostic Accuracy Studies 2 (QUADAS-2) tool[1]. Two reviewers would assess the studies separately, with the discrepancies solved by consensus.

1. DOMAIN 1 PATIENT SELECTION

1.1 RISK OF BIAS

Following data would be extracted:

(1) **Design**: whether the study was prospective or cross-sectional.

(2) **Settings**: where the study was conducted (in ICU, wards or emergency room etc.), the duration of study, and in which country the study was conducted.

(3) **Inclusion Criteria**: definition of the high-risk population.

(4) **Exclusion Criteria**: definition of the population not included in the study.

Answers to the questions:

(1) Was a consecutive or random sample of patients enrolled?

Refer to the data in “Design”. Fail to point out explicitly whether the patients were “consecutively” enrolled would be evaluated as risk of “Unclear”.

(2) Was a case-control design avoided?

Refer to the data in “Design”. A case-control study would be evaluated as “No”, in a study which patients enrolled were not explained explicitly as “consecutive”, the risk of “case-control” design was judged by the reviewers, with discrepancies solved by consensus.

(3) Did the study avoid inappropriate exclusion?

Refer to the data in “Exclusion Criteria”. Following reasons would be considered as appropriate exclusions: age < 16 years-old or > 80 years-old, immunocompromise (HIV-positive, glucocorticoids use, neutropenia, leukopenia, advanced malignancy), pregnancy or breastfeeding, refuse to participate, refuse to the therapy, discharged or die early (< 24 hours) after admission. Other reasons for exclusion would be evaluated as “Unclear”. Obvious wrong classification of exclusion would be evaluated as “No”. Comments would be given if assessed as “No”.

Could the selection of patients have introduced bias?

We defined “Yes” as 0 point, “Unclear” as 1 point and “No” as 2 points for each question above, with all the points added on for all three questions, we defined 0-1 point as “Low” risk, 2-4 points as “Unclear” risk and 5-6 points as “High” risk.

1.2 APPLICABILITY

Following data was extracted:

(1) **Infection**: prevalence of sepsis. The ratio of sepsis to high-risk population.

(2) **Sites**: the leading three sites of infections.

(3) **Microbiology**: the percentage and number of patients in whom the microbiological isolation was positive. The ratio (or number of patients) with Gram-positive and Gram-negative isolations, or other microorganisms.

Answer to the question:

Are there concerns that the included patients do not match the review question?

In some studies, healthy volunteers were also recruited as control group, if the index test was to discriminate sepsis from high-risk population, no bias was introduced; otherwise, if the index test was to discriminate sepsis from high-risk population and healthy volunteers, the risk of bias would be rated as “High”.

Articles without any description of at least one features of the infection listed above would be rated as risk of “Unclear”. No description of any characteristics of the infection would be assessed as risk of “High”.

2. DOMAIN 2 INDEX TEXT

2.1 RISK OF BIAS

Following data would be extracted:

(1) **Timing**: the time when the blood samples were obtained.

(2) **Storing**: the condition samples were stored (especially the temperature at which the samples were stored), and whether the assays were taken in batches.

(3) **Method**: the assay method (ELISA, immunoblots etc.) and the brand of assay kit.

(4) **Cut-off**: whether the cut-off was pre-specified or determined by the optimal area under curve (AUC).

Answers to the questions:

(1) Were the index test results interpreted without knowledge of the results of the reference standard?

Theoretically, the interpretation of the ELISA results needs no knowledge of the results of reference standard. The declaration of “blindness” was not mandatory in this section.

(2) if a threshold was used, was it pre-specified?

Refer to the data in “Cut-off”, if the cut-off was pre-specified in the method section, it was assessed as “Yes”, otherwise “No”.

Could the conduct or the interpretation of the index test have introduced bias?

We defined “Yes” as 0 point, “Unclear” as 1 point and “No” as 2 points for each question above, with all the points added up for all three questions, we defined 0-1 point as “Low” risk, 2 points as “Unclear” risk and 3-4 points as “High” risk.

2.2 APPLICABILITY

Answer to the question:

Are there concerns that the index test, its conduct, or interpretation differ from the review question?

Refer to the data extracted mentioned above. Biases would be introduced if the blood samples were obtained at an inappropriate time (e.g. late after the patients were admitted), storing (at inappropriate temperature, not analysed in batches), or with a wrong method. Fail to describe the essential steps in the measurement (lack the data in the entries we listed above) would lead to potential risk and would be rated as “Unclear” risk

3. DOMAIN 3 REFERENCE STANDARD

3.1 RISK OF BIAS

The detailed description of the decision of reference standard (sepsis) should include the following contents: (1) the diagnosis should be based on clinical evidences (1 point) including radiographic and/or laboratory results, (2) and also microbiological results (may not declared in the method section, could be described in the result section) (1 point); (3) the diagnosis should be determined by more than two clinicians or an expertise panel (1 point)(merely declaration that the diagnosis was determined by the attending doctor would not be adequate); (4) the decision-making should be blind to the results of the index test, which should be explicitly pointed out in the article.

Answer to the question:

(1) Is the reference standard likely to correctly classify the target condition?

We gave 1 point to each content above (except for the blindness) in the description the reference standard if mentioned, in total of 3 points, with 3 points as “Yes”, 1-2 points as “Unclear” and 0 point as “No”.

(2) Were the reference standard results interpreted without knowledge of the results of the index test

If the blindness was declared in the article “Yes”, otherwise “Unclear”.

Could the reference standard, its conduct, or its interpretation have introduced bias?

Another 1 point was given to the “blindness” of the study which makes up a total of 4 points,

with 0-1 point as “High” risk, 2 points as “Unclear” risk and 3-4 points as “Low” risk.

3.2 APPICABILITY

Answer to the question:

Are there concerns that the target condition as defined by the reference standard does not match the review question?

We defined “sepsis” according to guidelines of the SSC (2012)[2], as the systemic inflammatory response syndrome (SIRS) in addition with infections (as in most of the studies). However, the details of the diagnosis procedure were not available in the articles, nor did we acknowledge the correctness of the classifications, hence, the judges were made based on the descriptions of how the diagnosis was achieved, and were remained to the reviewers with discrepancies solved by consensus. We rated “High” risk if no description of diagnostic procedure was made; “Unclear” risk if it was incomplete or lacked essential elements; “Low” risk if it was in full detail.

4. DOMAIN 4 FLOW AND TIMING

4.1 RISK OF BIAS

Following data would be extracted:

(1) **Missing measurements**: The flow diagram would be referred, in aim to inspect any patients who did not receive the index test and/or reference standard or who were excluded from the 2×2 table.

(2) **Time interval**: the time point at when the blood samples were obtained, and the time point at when the diagnosis was made.

Answers to the questions:

(1) Was there an appropriate interval between index test and reference standard?

The time point between the index test (blood sample obtain) and reference standard was compared, if it was at the same time, then it would be rated as “Yes”; if the time-point of diagnosis was not declared (which was common in studies), it would be rated as “Unclear”; if obvious intervals between index test and reference standard could be observed, then it would be rated as “No”.

(2) Did all patients receive a reference standard?

Refer to the data extracted. If the number of patients included was consistent with that in analysis, then “Yes”, otherwise “No”.

(3) Did patients receive the same reference standard?

Although the definition of sepsis was SIRS in addition with infections, the determination of

infection is comprehensive which includes the clinical manifestations, laboratory and microbiological results, and even sometimes the response to the empirical antibiotics use. The infection could be obvious in some cases but insidious in others. We believe that the clinicians used different approaches to identify infections, which makes the reference standard not unique. Hence, in studies that microbiological isolation was positive in part of the patients, we rated as “No” since we though that other approaches were applied in determination of the infection.

In studies patients were only included with positive microbiological isolation, we rated as “Yes”, however, in this case we would also rate “No” to question 1 and 3 in **DOMAIN 1, RISA OF BIAS** part, since it would inappropriately miss infectious patient with negative microbiological isolations. If the author did not declare that only the patients with positive microbiological isolations were included, meanwhile, the number of patients was the same with the patients with positive microbiological isolations, we assessed this question as “Yes”. In cases the total number of positive isolation was not told (even if the individual isolation was described), we assessed it as “Unclear”.

We define “Yes” as 0 point, “Unclear” as 1 point and “No” as 2 points, and sum up all the points, with 0-2 points as “Low” risk, 3-5 as “Unclear” risk and 6-8 “High” risk.

All the studies would be assessed and rated, with discrepancies solved by consensus, a detailed assessment report was subsequently generated, and would be presented in as a bar graph.

References

[1] Whiting PF, Rutjes AW, Westwood ME, et al. QUADAS-2: a revised tool for the quality assessment of diagnostic accuracy studies. Ann Intern Med. 2011. 155(8): 529-36.

[2] Dellinger RP, Levy MM, Rhodes A, et al. Surviving Sepsis Campaign: international guidelines for management of severe sepsis and septic shock, 2012. Intensive Care Med. 2013. 39(2): 165-228.
