# Supplementary material for: Diagnostic value of serum soluble triggering expressed receptor on myeloid cells 1 (sTREM-1) in suspected sepsis: a meta-analysis
Source: BMC Immunol. 2020 Jan 13;21:2. doi: 10.1186/s12865-020-0332-x (PMC6958609; doi:10.1186/s12865-020-0332-x)
Supplement: Supplementary file 3 — Additional file 3. Detailed characteristics and quality assessment of the included studies. [file 12865_2020_332_MOESM3_ESM.pdf]

QUADAS-2

ID: **17** Author: Su L Year: 2013 Reviewer: **FP & JYX**

DOMAIN 1: PATIENT SELECTION

A. RISK OF BIAS

Describe methods of patient selection:

1. Design: prospective
2. Settings: respiratory ICU, surgical ICU and emergency ICU, Sept 2009 to Jul 2011, China
3. Inclusion: >= 2 criteria of SIRS with first 24hr in ICU
4. Exclusion: < 18yr, immunodeficiency, reduced polymorphonuclear granulocyte counts, died within 24hr after admission, refuse to participate, quit further treatment on their own will during the period of observation

Was a consecutive or random sample of patients enrolled?	Unclear
Was a case-control design avoided?	Yes
Did the study avoid inappropriate exclusions?:	Yes

Could the selection of patients have introduced bias? **Risk of bias:** Low

B. APPLICABILITY:

Describe included patients:

1. Infections: n = 130, sepsis/SIRS = 100/30, mortality = 43%, 43/100
2. Sites: Pulmonary (83%), post-operative (31%) and urinary tract (24%)
3. Microbiology: Gram-positive in 37 patients (37%), Gram-negative in 81 patients (81%), fungi in 62 patients (62%)

Do the included patients and setting match the question? **Concerns regarding applicability:** Low

DOMAIN 2: INDEX TEST

A. RISK OF BIAS

Describe the index test and how it was conducted and interpreted

1. Timing: within 24hr after admission, and in day 3, 5, 7, 10, and 14
2. Storing: at -80 degree
3. Methods: ELISA (Quantikine Human TREM-1 Immunoassay ELISA Kit, R & D Systems, Minneapolis, Minnesota, USA
4. Cut-off: optimized from ROC (64.4pg/mL, sensitivity/specificity = 0.91/0.896, AUC = 0.978)

Were the index test results interpreted without knowledge of the results of the reference standard?	Yes
If a threshold was used, was it pre-specified?	No

Could the conduct or interpretation of the index test have introduced bias? **Risk of bias:** Unclear

B. APPLICABILITY:

Is there concern that the index test, its conduct, or interpretation differ fromt he review question **Concerns regarding applicability:** Low

QUADAS-2

ID: **17** Author: Su L Year: 2013 Reviewer: **FP & JYX**

DOMAIN 3: REFERENCE STANDARD

A. RISK OF BIAS

Describe the reference standard and how it was conducted and interpreted::

1. the detailed description of determination of sepsis not reported
2. microbial isolations seen result section

Is the reference standard likely to correctly classify the target condition? Unclear

Were the reference standard results interpreted without knowledge of the results of the index test? Unclear

Could the reference standard, its conduct, or its interpretation have introduced bias? **Risk of bias:** High

B. APPLICABILITY:

Is there concern that the target condition as defined by the reference standard does not match the review question? **Concerns regarding applicability:** High

DOMAIN 4: FLOW AND TIMING

A. RISK OF BIAS

Describe any patients who did not receive the index test(s) and or reference standard or who were excluded from the 2x2 table (refer to flow diagram):

none

Describe the time interval and any interventions between index test(s) and reference standard:

1. blood samples gathered within 24hr after admission
2. the diagnosis of sepsis not mentioned
3. summed number of positive microbiological isolation unknown

Was there an appropriate interval between index test and reference standard? Unclear

Did all patients receive a reference standard? Yes

Did patients receive the same reference standard? Unclear

Were all patients included in the analysis? Yes

Could the patient flow have introduced bias? Low

QUADAS-2

ID:	3	Author:	Bayram H et a	Year:	2015	Reviewer:	FP & JYX

DOMAIN 1: PATIENT SELECTION

A. RISK OF BIAS

Describe methods of patient selection:

1. Design: prospective
2. Settings: hospitalized pats, Turkey
3. Inclusion: >=2 criteria of SIRS
4. Exclusion: immunodeficiency and/or malignancey, organ transplantation, taking corticosteroids > 1mg/kg per day, < 18yr, > 80yr.

Comment: only patients with positive microbiological isolation was included in the sepsis group which may inappropriately miss the infectious patients with negative microbiological isolations

Was a consecutive or random sample of patients enrolled?	No
Was a case-control design avoided?	Yes
Did the study avoid inappropriate exclusions?:	No

Could the selection of patients have introduced bias? **Risk of bias:** Unclear

B. APPLICABILITY:

Describe included patients:

1. Infections: n = 74, sepsis/SIRS = 33/41, mortality = 54.54%, 18/33
2. Sites: Respiratory tract (13, 39.4%), GI tract (8, 24.2%) and urinary tract (7, 21%)
3. Microbiology: Gram-positive (7, 21.2%), Gram-negative (20, 60.6%), poly-microbial (6, 18.2%)

Do the included patients and setting match the question? **Concerns regarding applicability:** Low

DOMAIN 2: INDEX TEST

A. RISK OF BIAS

Describe the index test and how it was conducted and interpreted

1. Timing: blood sample taken at day 0, 3, 4, 7 ,14 and 21
2. Storing: at -80 degree
3. Methods: Human TREM-1 ELISA, R&D Systems, USA
4. Cut-off: optimized by AUC (199.72pg/mL, sensitivity/specificity = 0.818/0.732, AUC = 0.826)

Were the index test results interpreted without knowledge of the results of the reference standard?	Yes
If a threshold was used, was it pre-specified?	No

Could the conduct or interpretation of the index test have introduced bias? **Risk of bias:** Unclear

B. APPLICABILITY:

Is there concern that the index test, its conduct, or interpretation differ fromt he review question **Concerns regarding applicability:** Low

QUADAS-2

ID:	3	Author:	Bayram H et a	Year:	2015	Reviewer:	FP & JYX

DOMAIN 3: REFERENCE STANDARD

A. RISK OF BIAS

Describe the reference standard and how it was conducted and interpreted::

1. patients were visited at regular intervals and assessed clinically (1 point)
2. microbial results listed in the results section (1 point)

Is the reference standard likely to correctly classify the target condition?	Unclear
Were the reference standard results interpreted without knowledge of the results of the index test?	Unclear
**Could the reference standard, its conduct, or its interpretation have introduced bias?** **Risk of bias:**	Unclear

B. APPLICABILITY:

**Is there concern that the target condition as defined by the reference standard does not match the review question?** **Concerns regarding applicability:**	Unclear

DOMAIN 4: FLOW AND TIMING

A. RISK OF BIAS

Describe any patients who did not receive the index test(s) and or reference standard or who were excluded from the 2x2 table (refer to flow diagram):

none

Describe the time interval and any interventions between index test(s) and reference standard:

1. blood sample taken at day 0
2. time of determine of sepsis not reported

Was there an appropriate interval between index test and reference standard?	Unclear
Did all patients receive a reference standard?	Yes
Did patients receive the same reference standard?	Yes
Were all patients included in the analysis?	Yes
**Could the patient flow have introduced bias?**	Low

QUADAS-2

ID:	19	Author:	Yang J et al	Year:	2014	Reviewer:	WC & JYX

DOMAIN 1: PATIENT SELECTION

A. RISK OF BIAS

Describe methods of patient selection:

1. Design: prospective
2. Settings: ICU, Sept 2012 to Sept 2013, China
3. Inclusion: age 18-80yr, onset =< 48hr, SIRS, mechanical ventilated, radiographic manifestation of newly or persistent effusions
4. Exclusion: > 80yr, immunocompromised (corticosteroids, bone marrow or organ transplantation), leukopenia or neutropenia, died or discharged within 12hr, HIV positive, hematologic malignancy, chronic organ dysfunction, thyroid or pancreatic cancer, multiple transfusions, infection sites other than lung

Was a consecutive or random sample of patients enrolled?	Unclear
Was a case-control design avoided?	Yes
Did the study avoid inappropriate exclusions?:	Unclear

Could the selection of patients have introduced bias? **Risk of bias:** Unclear

B. APPLICABILITY:

Describe included patients:

1. Infections: n = 70, sepsis/SIRS = 39/31, mortality = 38%, 15/39
2. Sites: pneumonia
3. Microbiology: not reported

Do the included patients and setting match the question? **Concerns regarding applicability:** Unclear

DOMAIN 2: INDEX TEST

A. RISK OF BIAS

Describe the index test and how it was conducted and interpreted

1. Timing: at day 1, 4 and 7 of admission in sepsis, at day 1 and 4 in SIRS
2. Storing: -70 degree till assay
3. Methods: ELISA (Westang Bio-technology Co., Ltd., Shanghai, China)
4. Cut-off: optmized by AUC (172.15pg/mL, sensitivity/specifity = 0.789/0.821, AUC = 0.796)

Were the index test results interpreted without knowledge of the results of the reference standard?	Yes
If a threshold was used, was it pre-specified?	No

Could the conduct or interpretation of the index test have introduced bias? **Risk of bias:** Unclear

B. APPLICABILITY:

Is there concern that the index test, its conduct, or interpretation differ fromt he review question **Concerns regarding applicability:** Low

QUADAS-2

ID:	19	Author:	Yang J et al	Year:	2014	Reviewer:	WC & JYX

DOMAIN 3: REFERENCE STANDARD

A. RISK OF BIAS

Describe the reference standard and how it was conducted and interpreted::

expertise panel composed of >=2 physicians from respiratory department, ICU and infectious disease departmant made the classification of SIRS and sepsis according to the SSC Guidelines (2012) and Comnunity-Acquired Pneumonia Diagnostic and Therapeutic Guidelines (2006)

Is the reference standard likely to correctly classify the target condition?	Yes
Were the reference standard results interpreted without knowledge of the results of the index test?	Unclear

Could the reference standard, its conduct, or its interpretation have introduced bias? **Risk of bias:** Low

B. APPLICABILITY:

Is there concern that the target condition as defined by the reference standard does not match the review question? **Concerns regarding applicability:** Low

DOMAIN 4: FLOW AND TIMING

A. RISK OF BIAS

Describe any patients who did not receive the index test(s) and or reference standard or who were excluded from the 2x2 table (refer to flow diagram):

none

Describe the time interval and any interventions between index test(s) and reference standard:

1. blood samples taken at the first day of admission
2. the time of determination of infection not reported

Was there an appropriate interval between index test and reference standard?	Unclear
Did all patients receive a reference standard?	Yes
Did patients receive the same reference standard?	No
Were all patients included in the analysis?	Yes

Could the patient flow have introduced bias? Unclear

QUADAS-2

ID:	1	Author:	Aksaray S et al	Year:	2016	Reviewer:	WC & SSM

DOMAIN 1: PATIENT SELECTION

A. RISK OF BIAS

Describe methods of patient selection:

1. Design: prospective consecutive
2. Settings: medical-surgical ICU, May 2013 to Jan 2014
3. Inclusion: >= 2 criteria of SIRS during first 24hr in the units
4. Exclusion: < 18yr, acquired immunodeficiency syndrome, neutropenia, died within 24hr after admission, elected not to participate, declined treatment during observation

Was a consecutive or random sample of patients enrolled?	Yes
Was a case-control design avoided?	Yes
Did the study avoid inappropriate exclusions?:	Yes

Could the selection of patients have introduced bias? **Risk of bias:** Low

B. APPLICABILITY:

Describe included patients:

1. Infection: n = 90, sepsis/SIRS = 52/38, mortality = 32.7%, 17/52
2. Sites: Lung (21, 40.3%) and blood (11, 21%)
3. Microbiology: blood culture positive in 38(73.3%) patients with sepsis, methicillin-resistant coagulase-negative Staphylococcus (36%), Escherichia coli (13%), and Acinetobacter baumannii (13%)

Do the included patients and setting match the question? **Concerns regarding applicability:** Low

DOMAIN 2: INDEX TEST

A. RISK OF BIAS

Describe the index test and how it was conducted and interpreted

1. Timing: blood sample taken within the first 24hr
2. Storing: -80 degree
3. Methods: ELISA, MyBioSource, Inc., San Diego, CA, USA
4. Cut-off: optimized by AUC [133pg/mL, sensitivity/specificity = 0.7115/0.7632, AUC = 0.78 (95% CI 0.69-0.86)]

Were the index test results interpreted without knowledge of the results of the reference standard?	Yes
If a threshold was used, was it pre-specified?	No

Could the conduct or interpretation of the index test have introduced bias? **Risk of bias:** Unclear

B. APPLICABILITY:

Is there concern that the index test, its conduct, or interpretation differ fromt he review question **Concerns regarding applicability:** Low

QUADAS-2

ID:	1	Author:	Aksaray S et al	Year:	2016	Reviewer:	WC & SSM

DOMAIN 3: REFERENCE STANDARD

A. RISK OF BIAS

Describe the reference standard and how it was conducted and interpreted::

1. Medical records (1 point) retrospectively evaluated and patients classified as sepsis or SIRS at the admission by two clinicians (1 point) blinded to the biomarker results.
2. Microbiology described in the result section

Is the reference standard likely to correctly classify the target condition?	Yes
Were the reference standard results interpreted without knowledge of the results of the index test?	Yes

Could the reference standard, its conduct, or its interpretation have introduced bias? **Risk of bias:** Low

B. APPLICABILITY:

Is there concern that the target condition as defined by the reference standard does not match the review question? **Concerns regarding applicability:** Low

DOMAIN 4: FLOW AND TIMING

A. RISK OF BIAS

Describe any patients who did not receive the index test(s) and or reference standard or who were excluded from the 2x2 table (refer to flow diagram):

none

Describe the time interval and any interventions between index test(s) and reference standard:

1. medical records retrospectively evaluated and pats classified as sepsis or SIRS at the admission by two clinicians blinded to the biomarker results.
2. blood samples within the first 24hr

Was there an appropriate interval between index test and reference standard?	Yes
Did all patients receive a reference standard?	Yes
Did patients receive the same reference standard?	No
Were all patients included in the analysis?	Yes

Could the patient flow have introduced bias? Low

QUADAS-2

ID:	4	Author:	Brenner T et a	Year:	2016	Reviewer:	WC & SSM

DOMAIN 1: PATIENT SELECTION

A. RISK OF BIAS

Describe methods of patient selection:

1. Design: re-analysis of prospective cohort
2. Settings: surgical intensive and post-operative area, Jun 2009, Germany
3. Inclusion: definition of septic shock according to International Sepsis Definition (2003), 30 post-operative control following
4. Exclusion: not reported

Was a consecutive or random sample of patients enrolled?	No
Was a case-control design avoided?	Unclear
Did the study avoid inappropriate exclusions?:	Unclear

Could the selection of patients have introduced bias? **Risk of bias:** High

B. APPLICABILITY:

Describe included patients:

1. Infection: n = 90, sepsis/non-sepsis = 60/30
2. Sites: lung (12, 20%), gastrointestinal tract (32, 53%), genitourinary tract (6, 10%) ;
3. Microbiology: Gram-positive (16, 26.7%), Gram-negative (16, 26.7%)

Do the included patients and setting match the question? **Concerns regarding applicability:** Low

DOMAIN 2: INDEX TEST

A. RISK OF BIAS

Describe the index test and how it was conducted and interpreted

1. Timing: blood sample collected in sepsis pats at sepsis onset, 24hr, 4 days, 7 days, 14 days and 28 days; in post-operative pats prior to surgery, immediately after surgery procedure and 24hr later; blood from the volenteers were collected once.
2. Storing: not reported
3. Method: ELISA (R&D Systems, Minneapolis, MN, USA)
4. Cut-off: optimized by AUC (30pg/mL, sensitivity/specificity = 0.983/0.9, AUC = 0.955)

Were the index test results interpreted without knowledge of the results of the reference standard?	Yes
If a threshold was used, was it pre-specified?	No

Could the conduct or interpretation of the index test have introduced bias? **Risk of bias:** Unclear

B. APPLICABILITY:

Is there concern that the index test, its conduct, or interpretation differ fromt he review question **Concerns regarding applicability:** Unclear

QUADAS-2

ID: **4** Author: Brenner T et a Year: 2016 Reviewer: **WC & SSM**

DOMAIN 3: REFERENCE STANDARD

A. RISK OF BIAS

Describe the reference standard and how it was conducted and interpreted::

1. definition of septic shock according to International Sepsis Definition (2003) (1 point)
2. Microbial results listed in the result section (1 point)

Is the reference standard likely to correctly classify the target condition?	Unclear
Were the reference standard results interpreted without knowledge of the results of the index test?	Unclear
**Could the reference standard, its conduct, or its interpretation have introduced bias?** **Risk of bias:**	Unclear

B. APPLICABILITY:

**Is there concern that the target condition as defined by the reference standard does not match the review question?** **Concerns regarding applicability:**	Unclear

DOMAIN 4: FLOW AND TIMING

A. RISK OF BIAS

Describe any patients who did not receive the index test(s) and or reference standard or who were excluded from the 2x2 table (refer to flow diagram):

none

Describe the time interval and any interventions between index test(s) and reference standard:

1. the blood sample were taken at admission
2. timt point of determination of sepsis not reported

Was there an appropriate interval between index test and reference standard?	Unclear
Did all patients receive a reference standard?	Yes
Did patients receive the same reference standard?	No
Were all patients included in the analysis?	Yes
**Could the patient flow have introduced bias?**	Unclear

QUADAS-2

ID:	13	Author:	Li Z et al	Year:	2016	Reviewer:	WC & JYX

DOMAIN 1: PATIENT SELECTION

A. RISK OF BIAS

Describe methods of patient selection:

1. Design: prospective
2. Settings: ICU, Jan 2014 to Jun 2015, China
3. Inclusion: >= 2 criteria of SIRS
4. Exclusion: age < 18yr, pregnancy, terminal stage of chronic hepatic or renal disease, advanced malignancy, thyroid disease and severe immunocompromise.

Was a consecutive or random sample of patients enrolled?	Unclear
Was a case-control design avoided?	Yes
Did the study avoid inappropriate exclusions?:	Unclear

Could the selection of patients have introduced bias? **Risk of bias:** Unclear

B. APPLICABILITY:

Describe included patients:

1. Infection: n = 80, sepsis/SIRS = 50/30, mortality = 30%, 15/50
2. Sites: lower respiratory tract (24), urinary tract (11), abdominal (7).
3. Microbiology: not reported

Do the included patients and setting match the question? **Concerns regarding applicability:** Unclear

DOMAIN 2: INDEX TEST

A. RISK OF BIAS

Describe the index test and how it was conducted and interpreted

1. Timing: first day upon admission
2. Storing: -80 degree
3. Methods: ELISA R & D
4. Cut-off: optimized by AUC (123.5pg/mL, sensitivity/specificity = 0.76/0.766, AUC = 0.864)

Were the index test results interpreted without knowledge of the results of the reference standard?	Yes
If a threshold was used, was it pre-specified?	No

Could the conduct or interpretation of the index test have introduced bias? **Risk of bias:** Unclear

B. APPLICABILITY:

Is there concern that the index test, its conduct, or interpretation differ fromt he review question **Concerns regarding applicability:** Low

QUADAS-2

ID:	13	Author:	Li Z et al	Year:	2016	Reviewer:	WC & JYX

DOMAIN 3: REFERENCE STANDARD

A. RISK OF BIAS

Describe the reference standard and how it was conducted and interpreted::

determined comprehensively by patients' clinical manifestations, infection foci, microbiological and radiographical results

Is the reference standard likely to correctly classify the target condition?	Unclear
Were the reference standard results interpreted without knowledge of the results of the index test?	Unclear

Could the reference standard, its conduct, or its interpretation have introduced bias? **Risk of bias:** Unclear

B. APPLICABILITY:

Is there concern that the target condition as defined by the reference standard does not match the review question? **Concerns regarding applicability:** Unclear

DOMAIN 4: FLOW AND TIMING

A. RISK OF BIAS

Describe any patients who did not receive the index test(s) and or reference standard or who were excluded from the 2x2 table (refer to flow diagram):

none

Describe the time interval and any interventions between index test(s) and reference standard:

1. first day upon admission
2. time of determination of sepsis not reported

Was there an appropriate interval between index test and reference standard?	Unclear
Did all patients receive a reference standard?	Yes
Did patients receive the same reference standard?	No
Were all patients included in the analysis?	Yes

Could the patient flow have introduced bias? Unclear

QUADAS-2

ID: **15** Author: Song X et al Year: 2017 Reviewer: **FP & JYX**

DOMAIN 1: PATIENT SELECTION

A. RISK OF BIAS

Describe methods of patient selection:

1. Design: prospective
2. Settings: department of gastrointestinal surgery, Oct 2014 to Oct 2015, China
3. Inclusion: underwent surgery with the diagnosis of an acute abdomen
4. Exclusion: pregnancy, a progressive fatal disease or immunosuppressive therapy, malignancy, other extra-abdominal infections

Was a consecutive or random sample of patients enrolled?	Unclear
Was a case-control design avoided?	Yes
Did the study avoid inappropriate exclusions?:	Unclear

Could the selection of patients have introduced bias? **Risk of bias:** Unclear

B. APPLICABILITY:

Describe included patients:

1. Infections: n = 128, sepsis/SIRS = 68/60, mortality = 21.4%, 12/68
2. Sites: intestinal fistula (16, 23.5%), gastric fistula (13, 19.1%), acute appendicitis (12, 17.6%), ileus (15, 22.1%) and intestinal perforation (12, 17.6%)
3. Microbiology: not reported

Do the included patients and setting match the question? **Concerns regarding applicability:** Unclear

DOMAIN 2: INDEX TEST

A. RISK OF BIAS

Describe the index test and how it was conducted and interpreted

1. Timing: within 24hr after hospitalization
2. Storing: not reported.
3. Method: ELISA, Quantikine Human TREM-1 Immunoassay ELISA Kit, R & D, Minneapolis, MN USA
4. Cut-off: optimized by AUC (113.06ng/mL, sensitivity/specificity = 0.8/0.76, AUC = 0.82)

Were the index test results interpreted without knowledge of the results of the reference standard?	Yes
If a threshold was used, was it pre-specified?	No

Could the conduct or interpretation of the index test have introduced bias? **Risk of bias:** Unclear

B. APPLICABILITY:

Is there concern that the index test, its conduct, or interpretation differ fromt he review question **Concerns regarding applicability:** Unclear

QUADAS-2

ID:	15	Author:	Song X et al	Year:	2017	Reviewer:	FP & JYX

DOMAIN 3: REFERENCE STANDARD

A. RISK OF BIAS

Describe the reference standard and how it was conducted and interpreted::

abdominal infection based on the American College of Chest Physicians/Society of Critical Care Medicine (ACCP/SCCM) Sepsis Directory
by microbiologial test

Is the reference standard likely to correctly classify the target condition?	Unclear
Were the reference standard results interpreted without knowledge of the results of the index test?	Unclear

Could the reference standard, its conduct, or its interpretation have introduced bias? **Risk of bias:** High

B. APPLICABILITY:

Is there concern that the target condition as defined by the reference standard does not match the review question? **Concerns regarding applicability:** High

DOMAIN 4: FLOW AND TIMING

A. RISK OF BIAS

Describe any patients who did not receive the index test(s) and or reference standard or who were excluded from the 2x2 table (refer to flow diagram):

none

Describe the time interval and any interventions between index test(s) and reference standard:

1. time interval between between indext test and reference standard unknown
2. microbiological results not reported

Was there an appropriate interval between index test and reference standard?	Unclear
Did all patients receive a reference standard?	Yes
Did patients receive the same reference standard?	Unclear
Were all patients included in the analysis?	Yes

Could the patient flow have introduced bias? Low

QUADAS-2

ID: **16** Author: Soud DEM et Year: 2011 Reviewer: **WC & SSM**

DOMAIN 1: PATIENT SELECTION

A. RISK OF BIAS

Describe methods of patient selection:

1. Design: prospective
2. Settings: emergency surgical department, ICU of anesthesia, Jan to Sept 2010, Egypt
3. Inclusion >= 2 criteria of SIRS
4. Exclusion age < 16yr or > 50yr, immunocompromise, leukopenia, neutropenia, burn, diabetic mellitus, discharged before completion of study (14 days) or failed to survive

Comments: exclusion of pats with diabetes and those dicharged before 14 days or failed to survive would potentially influence the diagnositc power of sTREM-1

Was a consecutive or random sample of patients enrolled?	Unclear
Was a case-control design avoided?	Yes
Did the study avoid inappropriate exclusions?:	Unclear

Could the selection of patients have introduced bias? **Risk of bias:** Unclear

B. APPLICABILITY:

Describe included patients:

1. Infection: n = 70, sepsis/SIRS = 19/51
2. Sites: Abdomen (31.6%), chest (26.3) and urinary (15.8%)
3. Microbiological: isolated in 19/70 patients, Gram-negative in 10 patients (52.63%), Gram-positive in 7 patients (36.84%), fungi in 2 patients (10.53%)

Do the included patients and setting match the question? **Concerns regarding applicability:** Low

DOMAIN 2: INDEX TEST

A. RISK OF BIAS

Describe the index test and how it was conducted and interpreted

1. Timing: not mentioned
2. Storing: at -20 degree
3. Methods: ELISA Quantikine Human TREM-1 immunoassay kit (R&D Systems, Minneapolis, MN)
4. Cut-off optimized by ROC (254pg/mL, sensitivity/specificity = 0.947/0.918)

Were the index test results interpreted without knowledge of the results of the reference standard?	Yes
If a threshold was used, was it pre-specified?	No

Could the conduct or interpretation of the index test have introduced bias? **Risk of bias:** Unclear

B. APPLICABILITY:

Is there concern that the index test, its conduct, or interpretation differ fromt he review question **Concerns regarding applicability:** Unclear

QUADAS-2

ID:	16	Author:	Soud DEM et	Year:	2011	Reviewer:	WC & SSM

DOMAIN 3: REFERENCE STANDARD

A. RISK OF BIAS

Describe the reference standard and how it was conducted and interpreted::

diagnosis of infection depends on the presence of SIRS, lab, (1 point) microbiological (1 point) tests and the treating doctors (0 point)

Is the reference standard likely to correctly classify the target condition?	Unclear
Were the reference standard results interpreted without knowledge of the results of the index test?	Unclear
**Could the reference standard, its conduct, or its interpretation have introduced bias?** **Risk of bias:**	Unclear

B. APPLICABILITY:

**Is there concern that the target condition as defined by the reference standard does not match the review question?** **Concerns regarding applicability:**	Unclear

DOMAIN 4: FLOW AND TIMING

A. RISK OF BIAS

Describe any patients who did not receive the index test(s) and or reference standard or who were excluded from the 2x2 table (refer to flow diagram):

none

Describe the time interval and any interventions between index test(s) and reference standard:

The interval between index test and reference standards not described

Was there an appropriate interval between index test and reference standard?	Unclear
Did all patients receive a reference standard?	Yes
Did patients receive the same reference standard?	No
Were all patients included in the analysis?	Yes
**Could the patient flow have introduced bias?**	Unclear

QUADAS-2

ID:	18	Author:	Wang H et al	Year:	2011	Reviewer:	FP & JYX

DOMAIN 1: PATIENT SELECTION

A. RISK OF BIAS

Describe methods of patient selection:

1. Design: prospective consecutive
2. Settings: ICU, May 2009 to Jul 2010, China
3. Inclusion: patients been diagnosed as severe sepsis or septic shock
4. Exclusion: newly admitted (< 24hr), cancer, severe trauma or major operation

Was a consecutive or random sample of patients enrolled?	Yes
Was a case-control design avoided?	Yes
Did the study avoid inappropriate exclusions?:	Unclear

Could the selection of patients have introduced bias? **Risk of bias:** Low

B. APPLICABILITY:

Describe included patients:

1. Infection: n = 56, sepsis/SIRS = 32/24, mortality = 34%, 11/32
2. Sites: not reported
3. Microbiology: not reported

Do the included patients and setting match the question? **Concerns regarding applicability:** Unclear

DOMAIN 2: INDEX TEST

A. RISK OF BIAS

Describe the index test and how it was conducted and interpreted

1. Timing: within 24hr after hospitalization
2. Storing: at -80 degree till assay.
3. Methods: ELISA R&D Company, United States
4. Cut-off: optimized by ROC (135pg/mL, sensitivity/specificity = 0.938/0.847, AUC = 0.935)

Were the index test results interpreted without knowledge of the results of the reference standard?	Yes
If a threshold was used, was it pre-specified?	No

Could the conduct or interpretation of the index test have introduced bias? **Risk of bias:** Unclear

B. APPLICABILITY:

Is there concern that the index test, its conduct, or interpretation differ fromt he review question **Concerns regarding applicability:** Low

QUADAS-2

ID:	18	Author:	Wang H et al	Year:	2011	Reviewer:	FP & JYX

DOMAIN 3: REFERENCE STANDARD

A. RISK OF BIAS

Describe the reference standard and how it was conducted and interpreted::

not reported

Is the reference standard likely to correctly classify the target condition?	No
Were the reference standard results interpreted without knowledge of the results of the index test?	Unclear

Could the reference standard, its conduct, or its interpretation have introduced bias? **Risk of bias:** High

B. APPLICABILITY:

Is there concern that the target condition as defined by the reference standard does not match the review question? **Concerns regarding applicability:** High

DOMAIN 4: FLOW AND TIMING

A. RISK OF BIAS

Describe any patients who did not receive the index test(s) and or reference standard or who were excluded from the 2x2 table (refer to flow diagram):

none

Describe the time interval and any interventions between index test(s) and reference standard:

1. blood sample taken within 24hr after hospitalization
2. diagnosis determine time not mentioned

Was there an appropriate interval between index test and reference standard?	Unclear
Did all patients receive a reference standard?	Yes
Did patients receive the same reference standard?	Unclear
Were all patients included in the analysis?	Yes

Could the patient flow have introduced bias? Low

QUADAS-2

ID:	5	Author:	Dong Y et al	Year:	2012	Reviewer:	WC & JYX

DOMAIN 1: PATIENT SELECTION

A. RISK OF BIAS

Describe methods of patient selection:

1. Design: prospective
2. Settings: emergency and medical ICU, May 2010 to Jul 2011, China
3. Inclusion: pats w/ SIRS, onset < 24hr, age 18-80yr
4. Exclusion: age > 80yr, immunocompromise, leukopenia or neutropenia, discharged or died < 12hr of admission, HIV positive

Was a consecutive or random sample of patients enrolled?	Unclear
Was a case-control design avoided?	Yes
Did the study avoid inappropriate exclusions?:	Yes

Could the selection of patients have introduced bias? **Risk of bias:** Low

B. APPLICABILITY:

Describe included patients:

1. Infections: n = 64, sepsis/SIRS = 43/21, mortality = 32.5%, 14/43
2. Sites: Respiratory (60.5%), abdominal (14%) and biliary tract (5%)
3. Microbiology: not reported.

Do the included patients and setting match the question? **Concerns regarding applicability:** Unclear

DOMAIN 2: INDEX TEST

A. RISK OF BIAS

Describe the index test and how it was conducted and interpreted

1. Timing: 24hr within recruitment, day 4 and 7
2. Storing: -80 degree till assays
3. Methods: ELISA, R&D, US
4. Cut-off: by Youden Index (95.9pg/mL, sensitivity/specificity = 0.767/0.905, AUC = 0.868 (95% CI 0.782-0.953))

Were the index test results interpreted without knowledge of the results of the reference standard?	Yes
If a threshold was used, was it pre-specified?	No

Could the conduct or interpretation of the index test have introduced bias? **Risk of bias:** Unclear

B. APPLICABILITY:

Is there concern that the index test, its conduct, or interpretation differ fromt he review question **Concerns regarding applicability:** Low

QUADAS-2

ID:	5	Author:	Dong Y et al	Year:	2012	Reviewer:	WC & JYX

DOMAIN 3: REFERENCE STANDARD

A. RISK OF BIAS

Describe the reference standard and how it was conducted and interpreted::

by clinical manifestations, infectious foci, pathogens, radiographic results

Is the reference standard likely to correctly classify the target condition?	Unclear
Were the reference standard results interpreted without knowledge of the results of the index test?	Unclear

Could the reference standard, its conduct, or its interpretation have introduced bias? **Risk of bias:** Unclear

B. APPLICABILITY:

Is there concern that the target condition as defined by the reference standard does not match the review question? **Concerns regarding applicability:** Unclear

DOMAIN 4: FLOW AND TIMING

A. RISK OF BIAS

Describe any patients who did not receive the index test(s) and or reference standard or who were excluded from the 2x2 table (refer to flow diagram):

none

Describe the time interval and any interventions between index test(s) and reference standard:

1. sample taken < 24hr of recruitment
2. diagnose time unknown

Was there an appropriate interval between index test and reference standard?	Unclear
Did all patients receive a reference standard?	Yes
Did patients receive the same reference standard?	Unclear
Were all patients included in the analysis?	Yes

Could the patient flow have introduced bias? Low

QUADAS-2

ID:	9	Author:	Gibot S et al	Year:	2012	Reviewer:	WC & SSM

DOMAIN 1: PATIENT SELECTION

A. RISK OF BIAS

Describe methods of patient selection:

1. Design: prospective consecutive
2. Settings: ICU, France
3. Inclusion: patients newly hospitalized
4. Exclusion: no

Was a consecutive or random sample of patients enrolled?	Yes
Was a case-control design avoided?	Yes
Did the study avoid inappropriate exclusions?:	Yes

Could the selection of patients have introduced bias? **Risk of bias:** Low

B. APPLICABILITY:

Describe included patients:

1. Infections: n = 300, sepsis/non-sepsis = 154/146, mortality = 26%, 40/154
2. Sites: Lung (49.4%), abdomen (12.3%) and Genitourinary (11%)
3. Microbiological: Positive microbiological documents in 88 (57%) pats, gram-postive (55%) and gram-negative (45%)

Do the included patients and setting match the question? **Concerns regarding applicability:** Low

DOMAIN 2: INDEX TEST

A. RISK OF BIAS

Describe the index test and how it was conducted and interpreted

1. Timing: within 12hr after admission
2. Storing: not reported
3. Methods: ELISA, Quantikine kit assay (R&D Systems, Minneapolis, MN)
4. Cut-off: Youden Index (755pg/mL, sensitivity/specificity = 0.532/0.863, AUC = 0.73)

Were the index test results interpreted without knowledge of the results of the reference standard?	Yes
If a threshold was used, was it pre-specified?	No

Could the conduct or interpretation of the index test have introduced bias? **Risk of bias:** Unclear

B. APPLICABILITY:

Is there concern that the index test, its conduct, or interpretation differ fromt he review question **Concerns regarding applicability:** Unclear

QUADAS-2

ID:	9	Author:	Gibot S et al	Year:	2012	Reviewer:	WC & SSM

DOMAIN 3: REFERENCE STANDARD

A. RISK OF BIAS

Describe the reference standard and how it was conducted and interpreted::

1. microbiologic test sent at admission when infection suspected
2. Two intensivists reviewed the medical records and classified the diagnosis indepently
3. Intensivists were masked to the value of the biomarker

Is the reference standard likely to correctly classify the target condition?	Yes
Were the reference standard results interpreted without knowledge of the results of the index test?	Yes

Could the reference standard, its conduct, or its interpretation have introduced bias? **Risk of bias:** Low

B. APPLICABILITY:

Is there concern that the target condition as defined by the reference standard does not match the review question? **Concerns regarding applicability:** Low

DOMAIN 4: FLOW AND TIMING

A. RISK OF BIAS

Describe any patients who did not receive the index test(s) and or reference standard or who were excluded from the 2x2 table (refer to flow diagram):

none

Describe the time interval and any interventions between index test(s) and reference standard:

1. blood sample taken <12hr after admission
2. Two intensivists classified the diagnosis at admission

Was there an appropriate interval between index test and reference standard?	Yes
Did all patients receive a reference standard?	Yes
Did patients receive the same reference standard?	No
Were all patients included in the analysis?	Yes

Could the patient flow have introduced bias? Low

QUADAS-2

ID: **12** Author: Li L et al Year: 2013 Reviewer: **FP & SSM**

DOMAIN 1: PATIENT SELECTION

A. RISK OF BIAS

Describe methods of patient selection:

1. Design: prospective consecutive
2. Settings: surgical ICU, Jan to Oct 2006, China
3. Inclusion: >= 2 criteria of SIRS w/ suspected infection
4. Exclusion: immunocompromise (corticosteroids, bone marrow or organ transplant, leukopenia, neutropenia, hematologic malignancy)

Was a consecutive or random sample of patients enrolled?	Yes
Was a case-control design avoided?	Yes
Did the study avoid inappropriate exclusions?:	Yes

Could the selection of patients have introduced bias? **Risk of bias:** Low

B. APPLICABILITY:

Describe included patients:

1. Infections: n = 52, sepsis/SIRS = 38/14, mortality = 48%, 25/52
2. Sites: not reported
3. Microbiological: 23 pats infected w/ bacteria, 2 pats w/ fungi, 11 pats w/ both bacteria and fungi; among 34 pats infected w/ bacteria, 14 w/bacillus, 20 w/ cocci

Do the included patients and setting match the question? **Concerns regarding applicability:** Unclear

DOMAIN 2: INDEX TEST

A. RISK OF BIAS

Describe the index test and how it was conducted and interpreted

1. Timing: Within 12hr after admission.
2. Storing: -80 degree till use
3. Method: ELISA R&D Systems, Minneapolis, MN
4. Cut-off: by optimal AUC (73.57pg/mL, sensitivity/specificity = 0.79/0.79, AUC = 0.82)

Were the index test results interpreted without knowledge of the results of the reference standard?	Yes
If a threshold was used, was it pre-specified?	No

Could the conduct or interpretation of the index test have introduced bias? **Risk of bias:** Unclear

B. APPLICABILITY:

Is there concern that the index test, its conduct, or interpretation differ fromt he review question **Concerns regarding applicability:** Low

QUADAS-2

ID:	12	Author:	Li L et al	Year:	2013	Reviewer:	FP & SSM

DOMAIN 3: REFERENCE STANDARD

A. RISK OF BIAS

Describe the reference standard and how it was conducted and interpreted::

1. Two intensivest retrospectively reviewed the medical records and classified the pats with sepsis and SIRS, blind to the plasma measures
2. Microbiological results listed in the result section

Is the reference standard likely to correctly classify the target condition?	Yes
Were the reference standard results interpreted without knowledge of the results of the index test?	Yes

Could the reference standard, its conduct, or its interpretation have introduced bias? **Risk of bias:** Low

B. APPLICABILITY:

Is there concern that the target condition as defined by the reference standard does not match the review question? **Concerns regarding applicability:** Low

DOMAIN 4: FLOW AND TIMING

A. RISK OF BIAS

Describe any patients who did not receive the index test(s) and or reference standard or who were excluded from the 2x2 table (refer to flow diagram):

none

Describe the time interval and any interventions between index test(s) and reference standard:

1. blood samples taken within 12hr after admission
2. diagnosis made at the admission

Was there an appropriate interval between index test and reference standard?	Yes
Did all patients receive a reference standard?	Yes
Did patients receive the same reference standard?	Unclear
Were all patients included in the analysis?	Yes

Could the patient flow have introduced bias? Low

QUADAS-2

ID:	8	Author:	Gibot S et al	Year:	2004	Reviewer:	FP & SSM

DOMAIN 1: PATIENT SELECTION

A. RISK OF BIAS

Describe methods of patient selection:

1. Design: prospective consecutive
2. Settings: medical ICU, Jun to Sept 2003, France
3. Inclusion: >= 2 criteria of SIRS, w/ suspected infection
4. Exclusion: > 80yr, immunocompromised (steroids, transplant, leukopenia, hematological malignant tumor or AIDS), die or discharged < 12hr, presented with total absence of anti-microbial treatment

Comments: patients excluded with total absence of anti-microbial treatment could potentially over-estimate diagnositic ability of sTREM-1

Was a consecutive or random sample of patients enrolled?	Yes
Was a case-control design avoided?	Yes
Did the study avoid inappropriate exclusions?:	No

Could the selection of patients have introduced bias? **Risk of bias:** Unclear

B. APPLICABILITY:

Describe included patients:

1. Infection n = 76, sepsis/SIRS = 47/29, mortality = 32%, 15/47
2. Sites: Respiratory (55%), abdomen (22%)
3. Microbiology: microbiologically proven in 40/47, 55% G-, 42% G+ and 3% fungal

Do the included patients and setting match the question? **Concerns regarding applicability:** Low

DOMAIN 2: INDEX TEST

A. RISK OF BIAS

Describe the index test and how it was conducted and interpreted

1. Timing: within 12hr within admission and study enrollment
2. Storing: at -80 degree for batch analysis
3. Methods: immunoblots
4. Cut-off: decided by ROC (60ng/mL, sensitivity 96%, specificity 89%, AUC = 0.97)

Were the index test results interpreted without knowledge of the results of the reference standard?	Yes
If a threshold was used, was it pre-specified?	No

Could the conduct or interpretation of the index test have introduced bias? **Risk of bias:** Unclear

B. APPLICABILITY:

Is there concern that the index test, its conduct, or interpretation differ fromt he review question **Concerns regarding applicability:** Low

QUADAS-2

ID:	8	Author:	Gibot S et al	Year:	2004	Reviewer:	FP & SSM

DOMAIN 3: REFERENCE STANDARD

A. RISK OF BIAS

Describe the reference standard and how it was conducted and interpreted::

1. microbiological tests taken routinely (1 point)
2. medical records reviewed retrospectively (1 point) and diagnosis decided independently by 2 intensivists (1 point)
3. two intensivists blinded (1 point) to sTREM-1 values

Is the reference standard likely to correctly classify the target condition?	Yes
Were the reference standard results interpreted without knowledge of the results of the index test?	Yes

Could the reference standard, its conduct, or its interpretation have introduced bias? **Risk of bias:** Low

B. APPLICABILITY:

Is there concern that the target condition as defined by the reference standard does not match the review question? **Concerns regarding applicability:** Low

DOMAIN 4: FLOW AND TIMING

A. RISK OF BIAS

Describe any patients who did not receive the index test(s) and or reference standard or who were excluded from the 2x2 table (refer to flow diagram):

none

Describe the time interval and any interventions between index test(s) and reference standard:

1. assay taken within 12hr after admission
2. microbiological tests taken routinely

Was there an appropriate interval between index test and reference standard?	Unclear
Did all patients receive a reference standard?	Yes
Did patients receive the same reference standard?	No
Were all patients included in the analysis?	Yes

Could the patient flow have introduced bias? Unclear

QUADAS-2

ID:	10	Author:	Kofoed K et al	Year:	2007	Reviewer:	FP & JYX

DOMAIN 1: PATIENT SELECTION

A. RISK OF BIAS

Describe methods of patient selection:

1. Design: prospective consecutive
2. Settings: department of infectious disease, infectious disease unit in medical emergency department, Feb 2005 to Feb 2006, Denmark
3. Inclusion: newly admittly (<24hr), >18yr, >=2 criteria of SIRS
4. Exclusion: >24hr of admission, no written consent, < 18yr, refusal to participate

Was a consecutive or random sample of patients enrolled?	Yes
Was a case-control design avoided?	Yes
Did the study avoid inappropriate exclusions?:	Yes

Could the selection of patients have introduced bias? **Risk of bias:** Low

B. APPLICABILITY:

Describe included patients:

1. Infection: n=151, sepsis/SIRS=117/34
2. Sites: Respiratory (60.4%), urinary tract (26%) and GI tract (17%)
3. Microbiology: detailed isolation of pathogen was listed in the article, 96 w/ bacteria, 16 w/ virus and 5 w/ parasite, clinical relevant pathogens isolated in 74/117 in first 7 days

Do the included patients and setting match the question? **Concerns regarding applicability:** Low

DOMAIN 2: INDEX TEST

A. RISK OF BIAS

Describe the index test and how it was conducted and interpreted

1.Timing: blood sample obtained at inclusion
2. Storing: -20 degree up to one week then transferred to -80 degree for later analysis
3. Method: Luminex multiplex assay (Luminex corp. Austin, TX, USA)
4. Cut-off: optimal cut-off determined by ROC and Youden Index (3.5μg/L, sensitivity 0.82 specificity 0.4, AUC = 0.61)

Were the index test results interpreted without knowledge of the results of the reference standard?	Yes
If a threshold was used, was it pre-specified?	No

Could the conduct or interpretation of the index test have introduced bias? **Risk of bias:** Unclear

B. APPLICABILITY:

Is there concern that the index test, its conduct, or interpretation differ fromt he review question **Concerns regarding applicability:** Low

QUADAS-2

ID: **10** Author: Kofoed K et al Year: 2007 Reviewer: **FP & JYX**

DOMAIN 3: REFERENCE STANDARD

A. RISK OF BIAS

Describe the reference standard and how it was conducted and interpreted::

1. sample taken at patients inclusion followed routine hospital procedures
2. diagnosis determined based on clinical findings, lab findings, microbiological findings, response to treatments, radiographic and other imaging procedures (2 points)
3. an expert panel consisting of two infectious disease sepecialists reviewed the medical records and decided the diagnosis at admission, indepently, with disgreement solved by consensus (1 points)
4. the panel was blind to sTREM-s values (1 point)

Is the reference standard likely to correctly classify the target condition?	Yes
Were the reference standard results interpreted without knowledge of the results of the index test?	Yes

Could the reference standard, its conduct, or its interpretation have introduced bias? **Risk of bias:** Low

B. APPLICABILITY:

Is there concern that the target condition as defined by the reference standard does not match the review question? **Concerns regarding applicability:** Low

DOMAIN 4: FLOW AND TIMING

A. RISK OF BIAS

Describe any patients who did not receive the index test(s) and or reference standard or who were excluded from the 2x2 table (refer to flow diagram):

none

Describe the time interval and any interventions between index test(s) and reference standard:

1. blood samples obtain at inclusion
2. the panel reviewed the medical records and make the diagnosis at admission
3. diagnosis based on microbiological, lab, clincial and radiographic findings

Was there an appropriate interval between index test and reference standard?	Yes
Did all patients receive a reference standard?	Yes
Did patients receive the same reference standard?	No
Were all patients included in the analysis?	Yes

Could the patient flow have introduced bias? Low

QUADAS-2

ID:	7	Author:	Giamarellos-B	Year:	2008	Reviewer:	FP & JYX

DOMAIN 1: PATIENT SELECTION

A. RISK OF BIAS

Describe methods of patient selection:

1. Design: prospective
2. Settings: ICU, two years from Jan 2004, Greece
3. Inclusion: >18yr, injury severity score (ISS) > 25, >=2 criteria of SIRS
4. Exclusion: neutropenia, HIV infection, steroids use
5. 10 patients ISS > 25 without SIRS as control

Was a consecutive or random sample of patients enrolled?	Unclear
Was a case-control design avoided?	Yes
Did the study avoid inappropriate exclusions?:	Yes

Could the selection of patients have introduced bias? **Risk of bias:** Low

B. APPLICABILITY:

Describe included patients:

1. Infection: n = 69, sepsis/SIRS = 43/26, mortality = 34.9%, 15/43
2. Sites: HAP (79%), acute pyelonephritis (7%) or primary gram-negative bacteremia (14%)
3. Microbiology: not reported

Do the included patients and setting match the question? **Concerns regarding applicability:** Unclear

DOMAIN 2: INDEX TEST

A. RISK OF BIAS

Describe the index test and how it was conducted and interpreted

1. Timing: blood obtained at admission, day 4, 7 and 15; as well as within 24hr after the diagnosis of any septic complications
2. Storing: not reported
3. Method: homemade enzyme immunoabsorbent assays
4. Cut-off: optimized by ROC (40pg/mL, sensitivity/specificity = 0.565/0.917, AUC = 0.708)

Were the index test results interpreted without knowledge of the results of the reference standard?	Yes
If a threshold was used, was it pre-specified?	No

Could the conduct or interpretation of the index test have introduced bias? **Risk of bias:** Unclear

B. APPLICABILITY:

Is there concern that the index test, its conduct, or interpretation differ fromt he review question **Concerns regarding applicability:** Unclear

QUADAS-2

ID:	7	Author:	Giamarellos-B	Year:	2008	Reviewer:	FP & JYX

DOMAIN 3: REFERENCE STANDARD

A. RISK OF BIAS

Describe the reference standard and how it was conducted and interpreted::

chest X-rays, blood cultures, tracheobronchial secretion culture and chest & abdomen CT scan if necessary (2 points)
Comments: the description of procedure of diagnosis not competent

Is the reference standard likely to correctly classify the target condition?	Unclear
Were the reference standard results interpreted without knowledge of the results of the index test?	Unclear

Could the reference standard, its conduct, or its interpretation have introduced bias? **Risk of bias:** Unclear

B. APPLICABILITY:

Is there concern that the target condition as defined by the reference standard does not match the review question? **Concerns regarding applicability:** Unclear

DOMAIN 4: FLOW AND TIMING

A. RISK OF BIAS

Describe any patients who did not receive the index test(s) and or reference standard or who were excluded from the 2x2 table (refer to flow diagram):

none

Describe the time interval and any interventions between index test(s) and reference standard:

1. sTREM-1 tests taken at admission
2. time interval between index tests and reference standards unknown
3. diagnosis based on microbiological and radiographic results

Was there an appropriate interval between index test and reference standard?	Unclear
Did all patients receive a reference standard?	Yes
Did patients receive the same reference standard?	No
Were all patients included in the analysis?	Yes

Could the patient flow have introduced bias? Unclear

QUADAS-2

ID: **14** Author: Rivera-Chavez Year: 2009 Reviewer: **WC & SSM**

DOMAIN 1: PATIENT SELECTION

A. RISK OF BIAS

Describe methods of patient selection:

1. Design: prospective
2. Settings: surgical ICU, in USA
3. Inclusion: >= 2 criteria of SIRS
4. Exclusion: immunocompromised, leukopenia, neutropenia, die or discharged < 24hr, do not resuscitation (DNR)

Comment: only patients with positive microbial cultures were included in the sepsis group

Was a consecutive or random sample of patients enrolled?	No
Was a case-control design avoided?	Yes
Did the study avoid inappropriate exclusions?:	No

Could the selection of patients have introduced bias? **Risk of bias:** High

B. APPLICABILITY:

Describe included patients:

1. Infection: n = 93, sepsis/SIRS = 56/37, mortality = 11%, 6/56
2. Sites: lung (60%), abdomen (13%) and blood (12%)
3. Microbiology: 28 (30%) patients w/ gram-negative isolation, 22 (23%) w/ gram-positive isolation, 6 (7%) w/ fungus

Do the included patients and setting match the question? **Concerns regarding applicability:** Low

DOMAIN 2: INDEX TEST

A. RISK OF BIAS

Describe the index test and how it was conducted and interpreted

1. Timing: sample obtained within 24-36hr after admission
2. Storing: stored -70 degree till analysis
3. Method: DuoSet enzyme-linked immunosorbent assay (R&D Systems, Minneapolis, MN)
4. Cut-off: optimized by AUC (230pg/mL, sensitivity/specificity = 0.98/0.91, AUC = 0.97)

Were the index test results interpreted without knowledge of the results of the reference standard?	Yes
If a threshold was used, was it pre-specified?	No

Could the conduct or interpretation of the index test have introduced bias? **Risk of bias:** Unclear

B. APPLICABILITY:

Is there concern that the index test, its conduct, or interpretation differ fromt he review question **Concerns regarding applicability:** Low

QUADAS-2

ID:	14	Author:	Rivera-Chavez	Year:	2009	Reviewer:	WC & SSM

DOMAIN 3: REFERENCE STANDARD

A. RISK OF BIAS

Describe the reference standard and how it was conducted and interpreted::

diagnosis base on the decision of the attending physician (0 point), bacteriological evidence of infection and the presence of SIRS, and the positive of microbial culture (2 point)

Is the reference standard likely to correctly classify the target condition?	Unclear
Were the reference standard results interpreted without knowledge of the results of the index test?	Unclear

Could the reference standard, its conduct, or its interpretation have introduced bias? **Risk of bias:** Unclear

B. APPLICABILITY:

Is there concern that the target condition as defined by the reference standard does not match the review question? **Concerns regarding applicability:** Unclear

DOMAIN 4: FLOW AND TIMING

A. RISK OF BIAS

Describe any patients who did not receive the index test(s) and or reference standard or who were excluded from the 2x2 table (refer to flow diagram):

none

Describe the time interval and any interventions between index test(s) and reference standard:

1. the timing of the diagnosis of infection not explicitly indicated, whereas the sample taken 24-36hr within admission
2. all the patients w/ sepsis had microbial isolations

Was there an appropriate interval between index test and reference standard?	Unclear
Did all patients receive a reference standard?	Yes
Did patients receive the same reference standard?	Yes
Were all patients included in the analysis?	Yes

Could the patient flow have introduced bias? Low

QUADAS-2

ID:	2	Author:	Barati M et al	Year:	2010	Reviewer:	FP & SSM

DOMAIN 1: PATIENT SELECTION

A. RISK OF BIAS

Describe methods of patient selection:

1. Design: prospective consecutive
2. Settings: medical and surgical ICU, Oct 2007 to Apr 2008, Iran
3. Inclusion: patients with SIRS
4. Exclusion: not mentioned

Was a consecutive or random sample of patients enrolled?	Yes
Was a case-control design avoided?	Yes
Did the study avoid inappropriate exclusions?:	Unclear

Could the selection of patients have introduced bias? **Risk of bias:** Low

B. APPLICABILITY:

Describe included patients:

1. Infection: n = 95, sepsis/SIRS = 52/43, 37 non-SIRS as control group
2. Sites: not reported
3. Microbiology: details not reported

Do the included patients and setting match the question? **Concerns regarding applicability:** High

DOMAIN 2: INDEX TEST

A. RISK OF BIAS

Describe the index test and how it was conducted and interpreted

1. Timing: upon admission to the ICU
2. Storing: -80 degree till assays
3. Method: quantitative sandwich enzyme immunoassay technique (Quantikine, R&D systems, Minneapolis, USA)
4. Cut-off: determined by optimal sensivity and specificity [725pg/mL, sensitivity/specificity = 0.7/0.6, AUC = 0.65 (95% CI 0.53-0.76)]

Were the index test results interpreted without knowledge of the results of the reference standard?	Yes
If a threshold was used, was it pre-specified?	No

Could the conduct or interpretation of the index test have introduced bias? **Risk of bias:** Unclear

B. APPLICABILITY:

Is there concern that the index test, its conduct, or interpretation differ fromt he review question **Concerns regarding applicability:** Low

QUADAS-2

ID:	2	Author:	Barati M et al	Year:	2010	Reviewer:	FP & SSM

DOMAIN 3: REFERENCE STANDARD

A. RISK OF BIAS

Describe the reference standard and how it was conducted and interpreted::

1. Patients classified as sepsis and SIRS by two intensivists (1 point), by clinical and lab data (1 point), blinded to the results of sTREM-1
2. Microbiological results were routinely collected (1 point)

Is the reference standard likely to correctly classify the target condition?	Yes
Were the reference standard results interpreted without knowledge of the results of the index test?	Yes

Could the reference standard, its conduct, or its interpretation have introduced bias? **Risk of bias:** Low

B. APPLICABILITY:

Is there concern that the target condition as defined by the reference standard does not match the review question? **Concerns regarding applicability:** Low

DOMAIN 4: FLOW AND TIMING

A. RISK OF BIAS

Describe any patients who did not receive the index test(s) and or reference standard or who were excluded from the 2x2 table (refer to flow diagram):

none

Describe the time interval and any interventions between index test(s) and reference standard:

1. index test at 24hr at admission
2. standard reference at 24hr at admission
3. number of patients with positive microbiological isolation unknown

Was there an appropriate interval between index test and reference standard?	Yes
Did all patients receive a reference standard?	Yes
Did patients receive the same reference standard?	Unclear
Were all patients included in the analysis?	Yes

Could the patient flow have introduced bias? Low

QUADAS-2

ID:	11	Author:	Latour-Peterz	Year:	2010	Reviewer:	WC & SSM

DOMAIN 1: PATIENT SELECTION

A. RISK OF BIAS

Describe methods of patient selection:

1. Design: prospective, not strictly consecutive
2. Settings: general ICU, Spain
3. Inclusion: >= 18yr, SIRS
4. Exclusion: informed consent form not signed, blood sample could not obtain

Was a consecutive or random sample of patients enrolled?	No
Was a case-control design avoided?	Yes
Did the study avoid inappropriate exclusions?:	Yes

Could the selection of patients have introduced bias? **Risk of bias:** Unclear

B. APPLICABILITY:

Describe included patients:

1. Infection: n = 114, sepsis/SIRS = 72/42, mortality = 37.5%, 27/72
2. Sites: respiratory (40%), abdominal-pelvic (21%) and urinary (12.5%)
3. Microbiology: not reported

Do the included patients and setting match the question? **Concerns regarding applicability:** Unclear

DOMAIN 2: INDEX TEST

A. RISK OF BIAS

Describe the index test and how it was conducted and interpreted

1. Timing: as soon as the detection of SIRS
2. Storing: -80 degree
3. Method: ELISA (R & D Systems, Inc., Minneapolis, MN)
4. Cut-off: optimized by ROC

Were the index test results interpreted without knowledge of the results of the reference standard?	Yes
If a threshold was used, was it pre-specified?	No

Could the conduct or interpretation of the index test have introduced bias? **Risk of bias:** Unclear

B. APPLICABILITY:

Is there concern that the index test, its conduct, or interpretation differ fromt he review question **Concerns regarding applicability:** Low

QUADAS-2

ID:	11	Author:	Latour-Peterz	Year:	2010	Reviewer:	WC & SSM

DOMAIN 3: REFERENCE STANDARD

A. RISK OF BIAS

Describe the reference standard and how it was conducted and interpreted::

1. clinical data, microbiological results and imaging (2 points)
2. by 2 investigators (1 point)
3. blind to sTREM-1 (1 point)

Is the reference standard likely to correctly classify the target condition?	Yes
Were the reference standard results interpreted without knowledge of the results of the index test?	Yes

Could the reference standard, its conduct, or its interpretation have introduced bias? **Risk of bias:** Low

B. APPLICABILITY:

Is there concern that the target condition as defined by the reference standard does not match the review question? **Concerns regarding applicability:** Low

DOMAIN 4: FLOW AND TIMING

A. RISK OF BIAS

Describe any patients who did not receive the index test(s) and or reference standard or who were excluded from the 2x2 table (refer to flow diagram):

none

Describe the time interval and any interventions between index test(s) and reference standard:

1. serum sample taken as soon as possible after detection of SIRS
2. timing of diagnosis of sepsis not reported

Was there an appropriate interval between index test and reference standard?	Unclear
Did all patients receive a reference standard?	Yes
Did patients receive the same reference standard?	Unclear
Were all patients included in the analysis?	Yes

Could the patient flow have introduced bias? Low

QUADAS-2

ID:	6	Author:	Gamez-Diaz L	Year:	2011	Reviewer:	WC & JYX

DOMAIN 1: PATIENT SELECTION

A. RISK OF BIAS

Describe methods of patient selection:

1. Design: cross-sectional study with prospective data
2. Settings: ED, Jun 2007 to Sept 2008, Colombia
3. Inclusion >= 18yr, within 24hr of ED admission, pats with possible sepsis syndrome: 1) suspected or confirmed infection, 2) fever of unknown origin, 3) delirium or any type of encephalopathy of unknown origin, or 4) acute hypotension not explained by hemorrhage, myocardial infarction, stroke, or heart failure.
4. Exclusion 1) refusal by the patients, their families, or the attending physician to be part of the study; 2) antimicrobial treatment received at another medical institution immediately before admission to the study; 3) medical decision to treat the patient ambulatory or in a different institution within 24 hours after admission; 4) homeless or inability of the patient to follow up; and 5) previous participation in the same study.

Was a consecutive or random sample of patients enrolled?	No
Was a case-control design avoided?	Yes
Did the study avoid inappropriate exclusions?:	Unclear

Could the selection of patients have introduced bias? **Risk of bias:** Unclear

B. APPLICABILITY:

Describe included patients:

1. Infection: n = 616, sepsis/non-sepsis = 405/211 (15 pats not available for analysis 9 pats in no-sepsis), mortality = 13.5%, 56/416
2. Sites: CAP (93, 22%), urinary tract (67, 16%) and soft tissue (16%)
3. Microbiological: microbiologic diagnosis confirmed in 185 (65, 44%) sepsis patients

Do the included patients and setting match the question? **Concerns regarding applicability:** Low

DOMAIN 2: INDEX TEST

A. RISK OF BIAS

Describe the index test and how it was conducted and interpreted

1. Timing: within 24hr of the first ED evaluation
2. Storing: -80 degree for later assay
3. Method: ELISA (Quantikine kit, human TREM-1 immunoassay, Cat. No. DTRM10, R&D Systems, Inc., Minneapolis, MN).
4.Cut-off: by optimal AUC (135pg/mL, sensitivity/specificity = 0.6/0.592, AUC = 0.6138)

Were the index test results interpreted without knowledge of the results of the reference standard?	Yes
If a threshold was used, was it pre-specified?	No

Could the conduct or interpretation of the index test have introduced bias? **Risk of bias:** Unclear

B. APPLICABILITY:

Is there concern that the index test, its conduct, or interpretation differ fromt he review question **Concerns regarding applicability:** Low

QUADAS-2

ID: **6** Author: Gamez-Diaz L Year: 2011 Reviewer: **WC & JYX**

DOMAIN 3: REFERENCE STANDARD

A. RISK OF BIAS

Describe the reference standard and how it was conducted and interpreted::

1. sepsis defind by 3 experts consensus
2. by clincial, microbiological and laboratory results
3. blind to the results of sTREM-1

Is the reference standard likely to correctly classify the target condition?	Yes
Were the reference standard results interpreted without knowledge of the results of the index test?	Yes

Could the reference standard, its conduct, or its interpretation have introduced bias? **Risk of bias:** Low

B. APPLICABILITY:

Is there concern that the target condition as defined by the reference standard does not match the review question? **Concerns regarding applicability:** Low

DOMAIN 4: FLOW AND TIMING

A. RISK OF BIAS

Describe any patients who did not receive the index test(s) and or reference standard or who were excluded from the 2x2 table (refer to flow diagram):

15 pats did not have samples available for analysis

Describe the time interval and any interventions between index test(s) and reference standard:

1. blood sample taken within 24hr of the first ED evaluation
2. diagnosis time in the first 7 days

Was there an appropriate interval between index test and reference standard?	Unclear
Did all patients receive a reference standard?	Yes
Did patients receive the same reference standard?	No
Were all patients included in the analysis?	No

Could the patient flow have introduced bias? Unclear
