# Supplementary material for: Diagnostic value of serum soluble triggering expressed receptor on myeloid cells 1 (sTREM-1) in suspected sepsis: a meta-analysis
Source: BMC Immunol. 2020 Jan 13;21:2. doi: 10.1186/s12865-020-0332-x (PMC6958609; doi:10.1186/s12865-020-0332-x)
Supplement: Supplementary file 4 — Additional file 4. Sub-group analysis according to the cut-off values. [file 12865_2020_332_MOESM4_ESM.docx]

**Additional file 4 Sub-group analysis by cut-offs**

| Studies | Cut-off Ranges (pg/mL) | Subjects No. | AuROC (95% CI) | Sensitivity (95% CI) | Specificity (95% CI) | PLR (95% CI) | NLR (95% CI) | DOR (95% CI) |
| --- | --- | --- | --- | --- | --- | --- | --- | --- |
| [24] [26] [27] [28] [29] [32] [35] [36] [38] [40] [41] [42] | 30-199.72 | 1519 | 0.87 (0.84-0.90) | 0.81 (0.73- 0.87) | 0.80 (0.73- 0.86) | 4.1 (2.8- 5.9) | 0.24 (0.16- 0.36) | 17 (8-36) |
| [25] [30] [31] [33] [34] [37] [39] | 230-600000 | 899 | 0.89 (0.86-0.91) | 0.85 (0.64- 0.95) | 0.80 (0.65-0.90) | 4.3 (2.1- 8.9) | 0.19 (0.07- 0.54) | 23 (5-114) |
