# Supplementary figures and images for: Diagnostic value of serum soluble triggering expressed receptor on myeloid cells 1 (sTREM-1) in suspected sepsis: a meta-analysis
Source: BMC Immunol. 2020 Jan 13;21:2. doi: 10.1186/s12865-020-0332-x (PMC6958609; doi:10.1186/s12865-020-0332-x)

# A

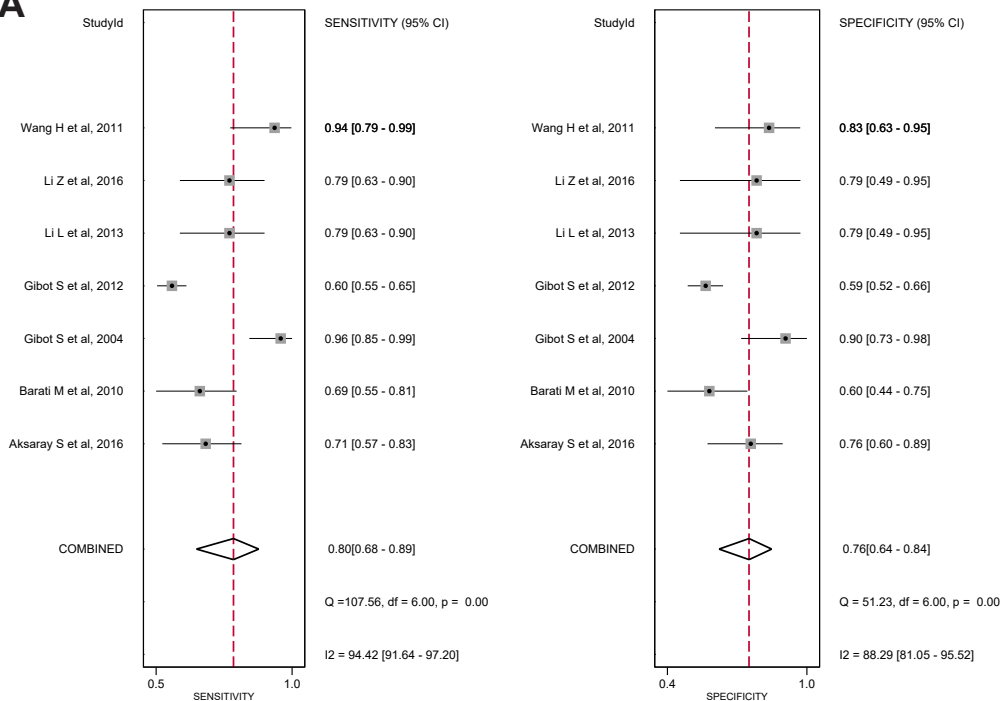

# B

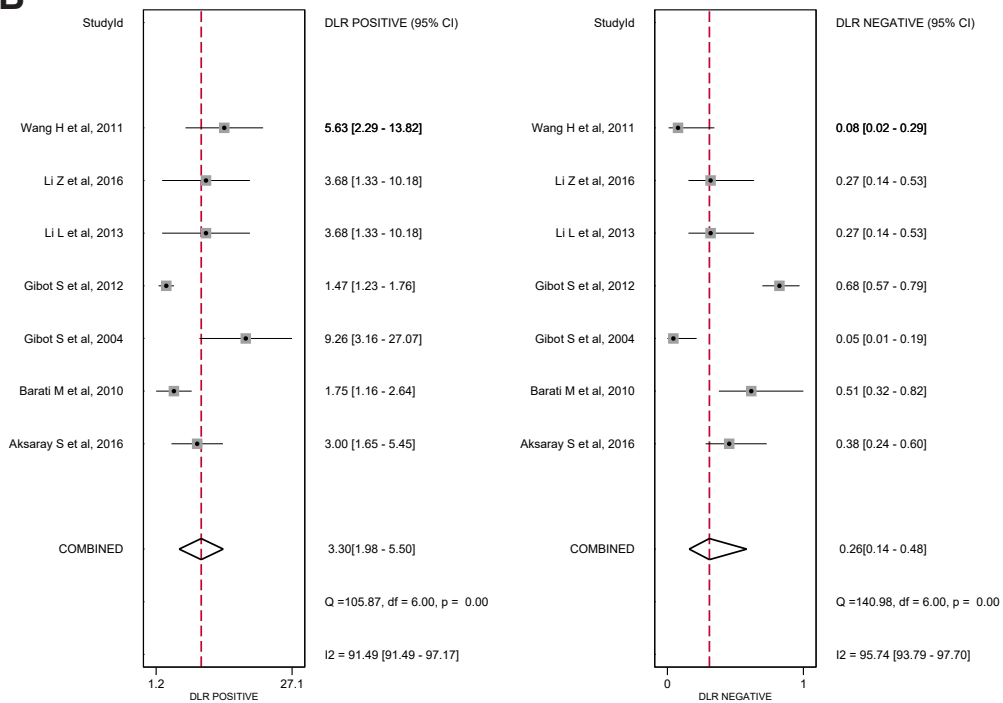

Supplement: Supplementary file 5 — Additional file 5: Figure S1. Sub-group analysis of studies conducted in ICU with patients consecutively recruited. The sub-group of 7 prospective trials conducted in the ICU, in which the patients with SIRS were consecutively recruited. A. Forest plots showing the sensitivity (0.80, 95% CI 0.68–0.89) and specificity (0.76, 95% CI 0.64–0.84) of sTREM-1; B. Forest plots showing the positive diagnostic likelihood ratio (DLR positive) (3.30, 95% CI 1.98–5.50) and negative diagnostic likelihood ratio (DLR negative) (0.26, 95% CI 0.14–0.48) of sTREM-1. [file 12865_2020_332_MOESM5_ESM.pdf]
