# Supplementary material for: Diagnostic value of serum soluble triggering expressed receptor on myeloid cells 1 (sTREM-1) in suspected sepsis: a meta-analysis
Source: BMC Immunol. 2020 Jan 13;21:2. doi: 10.1186/s12865-020-0332-x (PMC6958609; doi:10.1186/s12865-020-0332-x)
Supplement: Supplementary file 6 — Additional file 6: Figure S2. Sub-group analyses according to the meta-analysis results. [file 12865_2020_332_MOESM6_ESM.pdf]

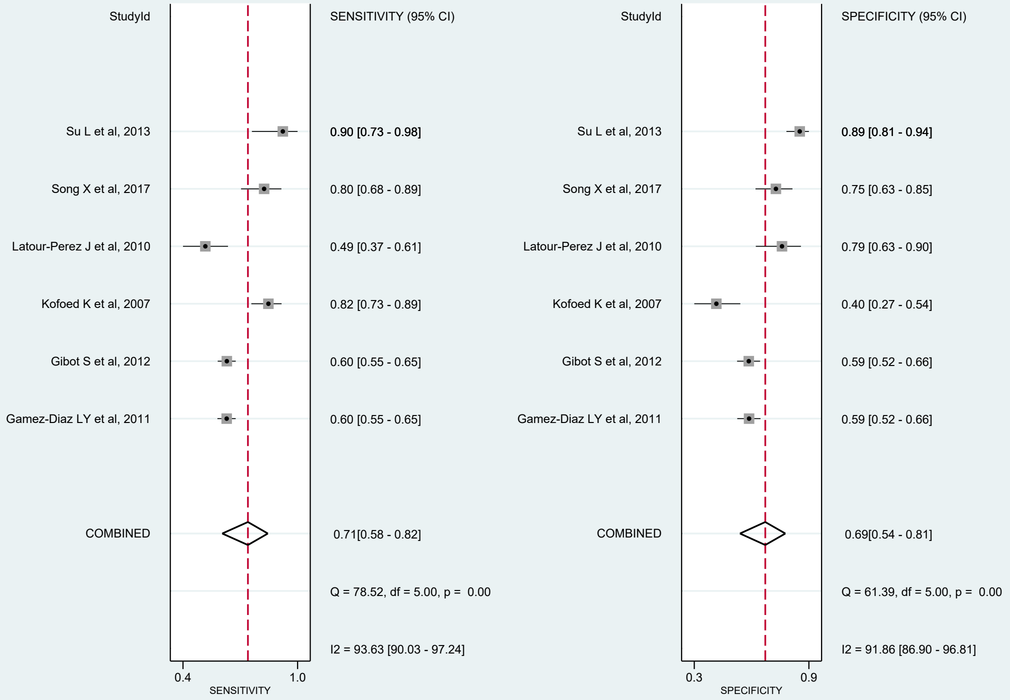

StudyId
SENSITIVITY (95% CI)
Su L et al, 2013
0.90 [0.73 - 0.98]
Song X et al, 2017
0.80 [0.68 - 0.89]
Latour-Perez J et al, 2010
0.49 [0.37 - 0.61]
Kofoed K et al, 2007
0.82 [0.73 - 0.89]
Gibot S et al, 2012
0.60 [0.55 - 0.65]
Gamez-Diaz LY et al, 2011
0.60 [0.55 - 0.65]
COMBINED
0.71[0.58 - 0.82]
Q = 78.52, df = 5.00, p = 0.00
I2 = 93.63 [90.03 - 97.24]
0.4
1.0
SENSITIVITY
StudyId
SPECIFICITY (95% CI)
Su L et al, 2013
0.89 [0.81 - 0.94]
Song X et al, 2017
0.75 [0.63 - 0.85]
Latour-Perez J et al, 2010
0.79 [0.63 - 0.90]
Kofoed K et al, 2007
0.40 [0.27 - 0.54]
Gibot S et al, 2012
0.59 [0.52 - 0.66]
Gamez-Diaz LY et al, 2011
0.59 [0.52 - 0.66]
COMBINED
0.69[0.54 - 0.81]
Q = 61.39, df = 5.00, p = 0.00
I2 = 91.86 [86.90 - 96.81]
0.3
0.9
SPECIFICITY

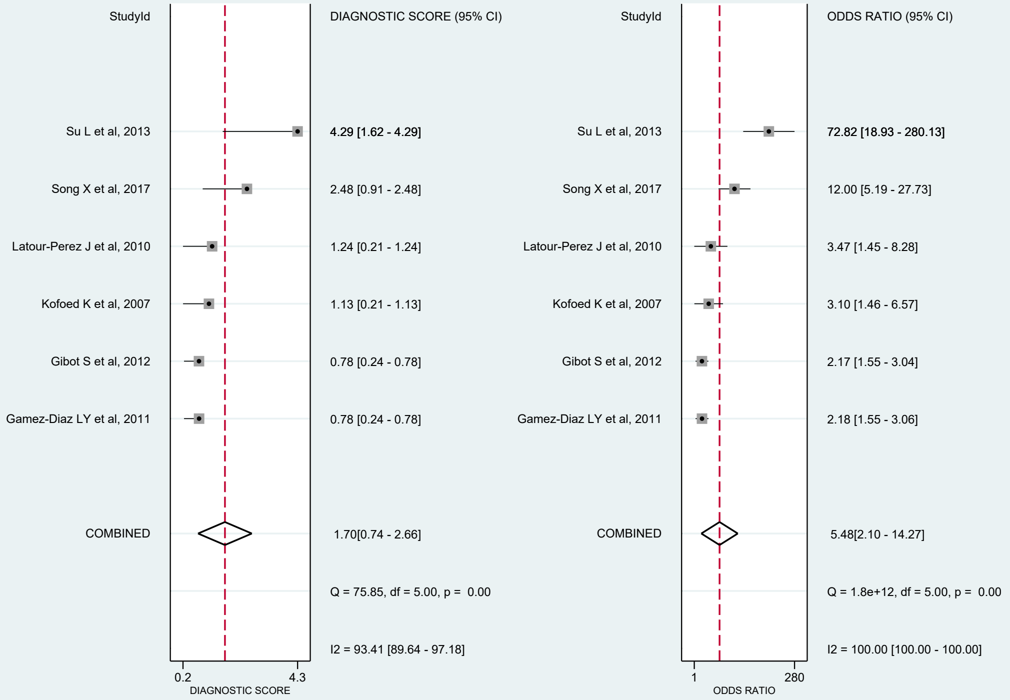
StudyId
DIAGNOSTIC SCORE (95% CI)
Su L et al, 2013
4.29 [1.62 - 4.29]
Song X et al, 2017
2.48 [0.91 - 2.48]
Latour-Perez J et al, 2010
1.24 [0.21 - 1.24]
Kofoed K et al, 2007
1.13 [0.21 - 1.13]
Gibot S et al, 2012
0.78 [0.24 - 0.78]
Gamez-Diaz LY et al, 2011
0.78 [0.24 - 0.78]
COMBINED
1.70[0.74 - 2.66]
Q = 75.85, df = 5.00, p = 0.00
I2 = 93.41 [89.64 - 97.18]
0.2
4.3
DIAGNOSTIC SCORE
StudyId
ODDS RATIO (95% CI)
Su L et al, 2013
72.82 [18.93 - 280.13]
Song X et al, 2017
12.00 [5.19 - 27.73]
Latour-Perez J et al, 2010
3.47 [1.45 - 8.28]
Kofoed K et al, 2007
3.10 [1.46 - 6.57]
Gibot S et al, 2012
2.17 [1.55 - 3.04]
Gamez-Diaz LY et al, 2011
2.18 [1.55 - 3.06]
COMBINED
5.48[2.10 - 14.27]
Q = 1.8e+12, df = 5.00, p = 0.00
I2 = 100.00 [100.00 - 100.00]
1
280
ODDS RATIO

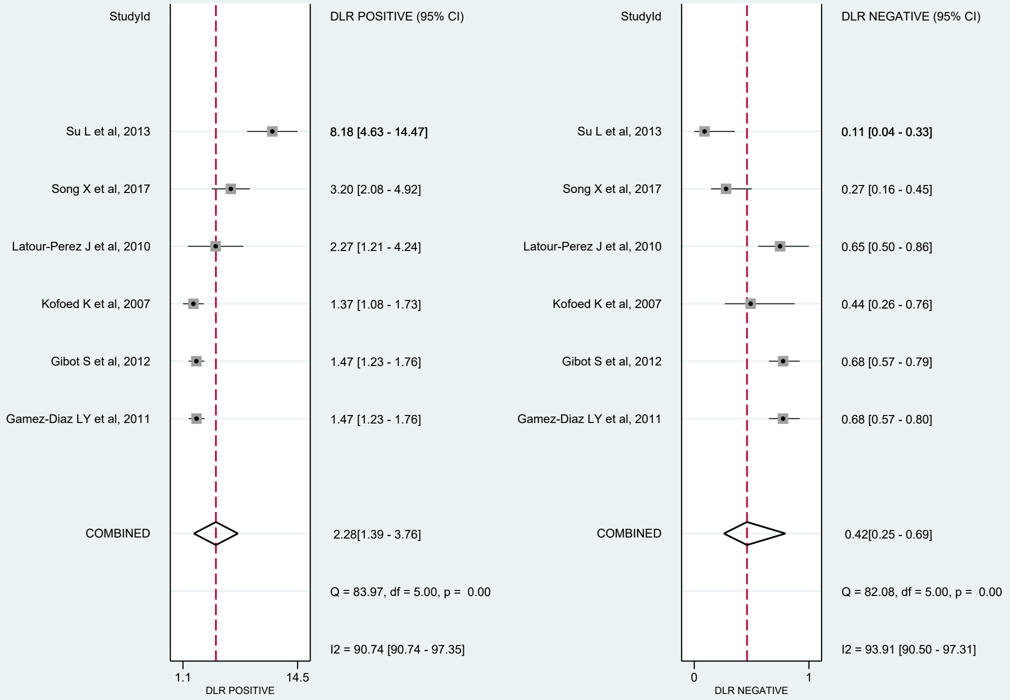
StudyId
DLR POSITIVE (95% CI)
Su L et al, 2013
8.18 [4.63 - 14.47]
Song X et al, 2017
3.20 [2.08 - 4.92]
Latour-Perez J et al, 2010
2.27 [1.21 - 4.24]
Kofoed K et al, 2007
1.37 [1.08 - 1.73]
Gibot S et al, 2012
1.47 [1.23 - 1.76]
Gamez-Diaz LY et al, 2011
1.47 [1.23 - 1.76]
COMBINED
2.28[1.39 - 3.76]
Q = 83.97, df = 5.00, p = 0.00
I2 = 90.74 [90.74 - 97.35]
1.1
14.5
DLR POSITIVE
StudyId
DLR NEGATIVE (95% CI)
Su L et al, 2013
0.11 [0.04 - 0.33]
Song X et al, 2017
0.27 [0.16 - 0.45]
Latour-Perez J et al, 2010
0.65 [0.50 - 0.86]
Kofoed K et al, 2007
0.44 [0.26 - 0.76]
Gibot S et al, 2012
0.68 [0.57 - 0.79]
Gamez-Diaz LY et al, 2011
0.68 [0.57 - 0.80]
COMBINED
0.42[0.25 - 0.69]
Q = 82.08, df = 5.00, p = 0.00
I2 = 93.91 [90.50 - 97.31]
0
1
DLR NEGATIVE

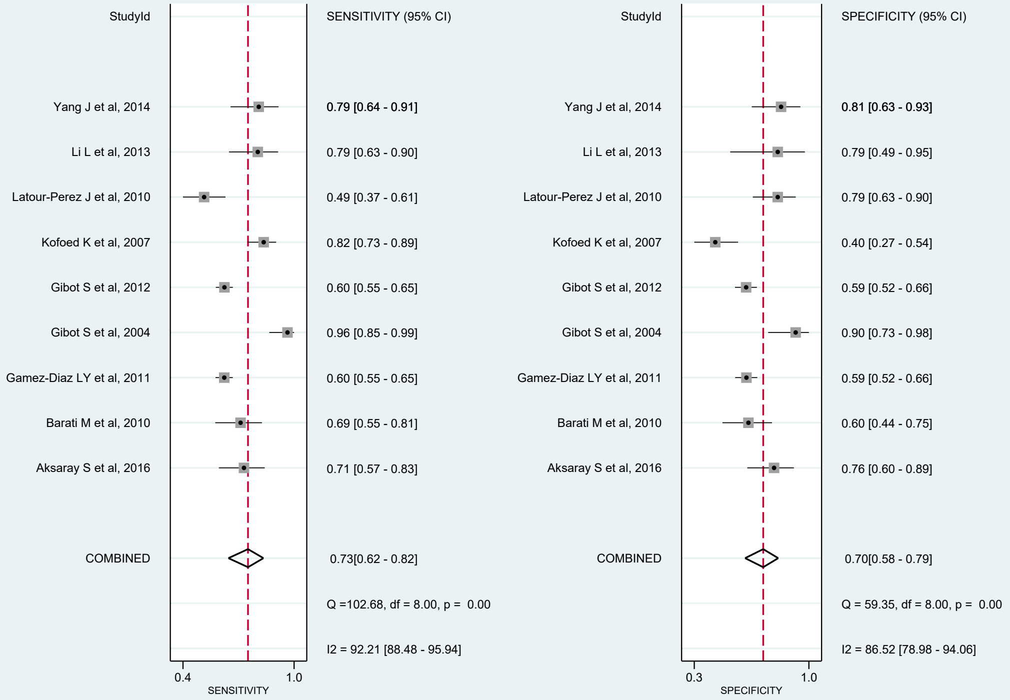

StudyId
SENSITIVITY (95% CI)
Yang J et al, 2014
0.79 [0.64 - 0.91]
Li L et al, 2013
0.79 [0.63 - 0.90]
Latour-Perez J et al, 2010
0.49 [0.37 - 0.61]
Kofoed K et al, 2007
0.82 [0.73 - 0.89]
Gibot S et al, 2012
0.60 [0.55 - 0.65]
Gibot S et al, 2004
0.96 [0.85 - 0.99]
Gamez-Diaz LY et al, 2011
0.60 [0.55 - 0.65]
Barati M et al, 2010
0.69 [0.55 - 0.81]
Aksaray S et al, 2016
0.71 [0.57 - 0.83]
COMBINED
0.73[0.62 - 0.82]
Q =102.68, df = 8.00, p = 0.00
I2 = 92.21 [88.48 - 95.94]
0.4
1.0
SENSITIVITY
StudyId
SPECIFICITY (95% CI)
Yang J et al, 2014
0.81 [0.63 - 0.93]
Li L et al, 2013
0.79 [0.49 - 0.95]
Latour-Perez J et al, 2010
0.79 [0.63 - 0.90]
Kofoed K et al, 2007
0.40 [0.27 - 0.54]
Gibot S et al, 2012
0.59 [0.52 - 0.66]
Gibot S et al, 2004
0.90 [0.73 - 0.98]
Gamez-Diaz LY et al, 2011
0.59 [0.52 - 0.66]
Barati M et al, 2010
0.60 [0.44 - 0.75]
Aksaray S et al, 2016
0.76 [0.60 - 0.89]
COMBINED
0.70[0.58 - 0.79]
Q = 59.35, df = 8.00, p = 0.00
I2 = 86.52 [78.98 - 94.06]
0.3
1.0
SPECIFICITY

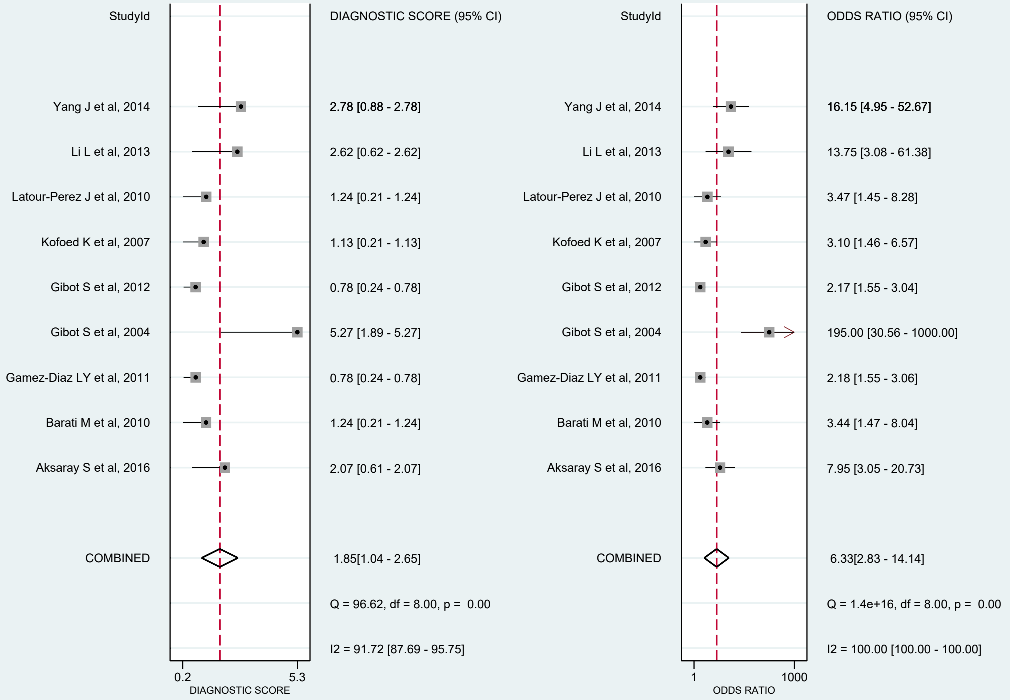

StudyId
DIAGNOSTIC SCORE (95% CI)
Yang J et al, 2014
2.78 [0.88 - 2.78]
Li L et al, 2013
2.62 [0.62 - 2.62]
Latour-Perez J et al, 2010
1.24 [0.21 - 1.24]
Kofoed K et al, 2007
1.13 [0.21 - 1.13]
Gibot S et al, 2012
0.78 [0.24 - 0.78]
Gibot S et al, 2004
5.27 [1.89 - 5.27]
Gamez-Diaz LY et al, 2011
0.78 [0.24 - 0.78]
Barati M et al, 2010
1.24 [0.21 - 1.24]
Aksaray S et al, 2016
2.07 [0.61 - 2.07]
COMBINED
1.85[1.04 - 2.65]
Q = 96.62, df = 8.00, p = 0.00
I2 = 91.72 [87.69 - 95.75]
0.2
5.3
DIAGNOSTIC SCORE
StudyId
ODDS RATIO (95% CI)
Yang J et al, 2014
16.15 [4.95 - 52.67]
Li L et al, 2013
13.75 [3.08 - 61.38]
Latour-Perez J et al, 2010
3.47 [1.45 - 8.28]
Kofoed K et al, 2007
3.10 [1.46 - 6.57]
Gibot S et al, 2012
2.17 [1.55 - 3.04]
Gibot S et al, 2004
195.00 [30.56 - 1000.00]
Gamez-Diaz LY et al, 2011
2.18 [1.55 - 3.06]
Barati M et al, 2010
3.44 [1.47 - 8.04]
Aksaray S et al, 2016
7.95 [3.05 - 20.73]
COMBINED
6.33[2.83 - 14.14]
Q = 1.4e+16, df = 8.00, p = 0.00
I2 = 100.00 [100.00 - 100.00]
1
1000
ODDS RATIO

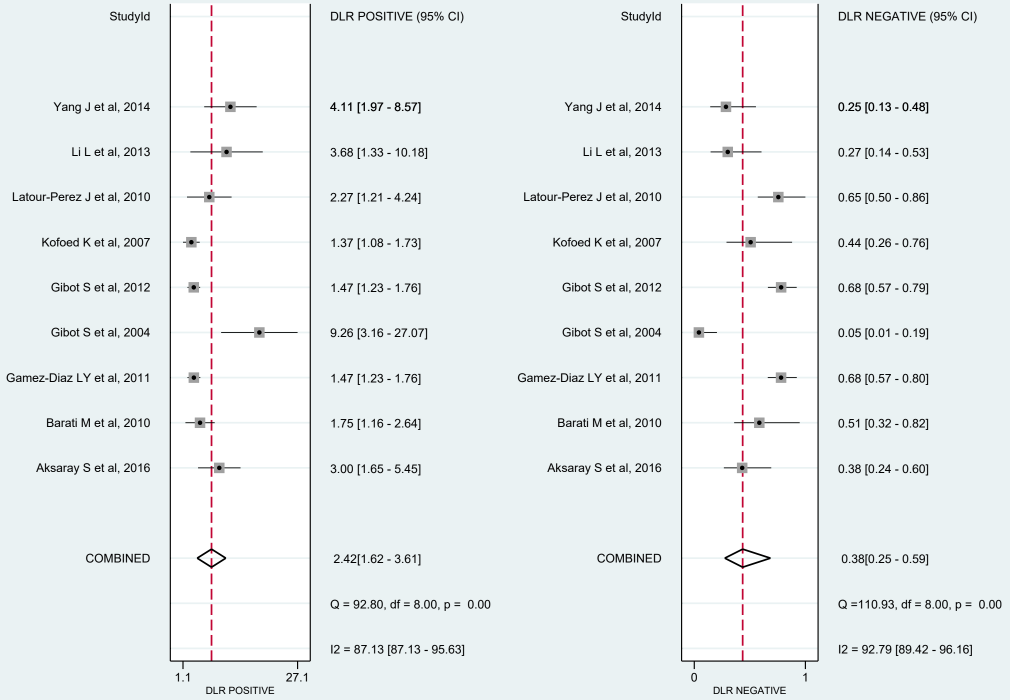
StudyId
DLR POSITIVE (95% CI)
Yang J et al, 2014
4.11 [1.97 - 8.57]
Li L et al, 2013
3.68 [1.33 - 10.18]
Latour-Perez J et al, 2010
2.27 [1.21 - 4.24]
Kofoed K et al, 2007
1.37 [1.08 - 1.73]
Gibot S et al, 2012
1.47 [1.23 - 1.76]
Gibot S et al, 2004
9.26 [3.16 - 27.07]
Gamez-Diaz LY et al, 2011
1.47 [1.23 - 1.76]
Barati M et al, 2010
1.75 [1.16 - 2.64]
Aksaray S et al, 2016
3.00 [1.65 - 5.45]
COMBINED
2.42[1.62 - 3.61]
Q = 92.80, df = 8.00, p = 0.00
I2 = 87.13 [87.13 - 95.63]
1.1
27.1
DLR POSITIVE
StudyId
DLR NEGATIVE (95% CI)
Yang J et al, 2014
0.25 [0.13 - 0.48]
Li L et al, 2013
0.27 [0.14 - 0.53]
Latour-Perez J et al, 2010
0.65 [0.50 - 0.86]
Kofoed K et al, 2007
0.44 [0.26 - 0.76]
Gibot S et al, 2012
0.68 [0.57 - 0.79]
Gibot S et al, 2004
0.05 [0.01 - 0.19]
Gamez-Diaz LY et al, 2011
0.68 [0.57 - 0.80]
Barati M et al, 2010
0.51 [0.32 - 0.82]
Aksaray S et al, 2016
0.38 [0.24 - 0.60]
COMBINED
0.38[0.25 - 0.59]
Q =110.93, df = 8.00, p = 0.00
I2 = 92.79 [89.42 - 96.16]
0
1
DLR NEGATIVE

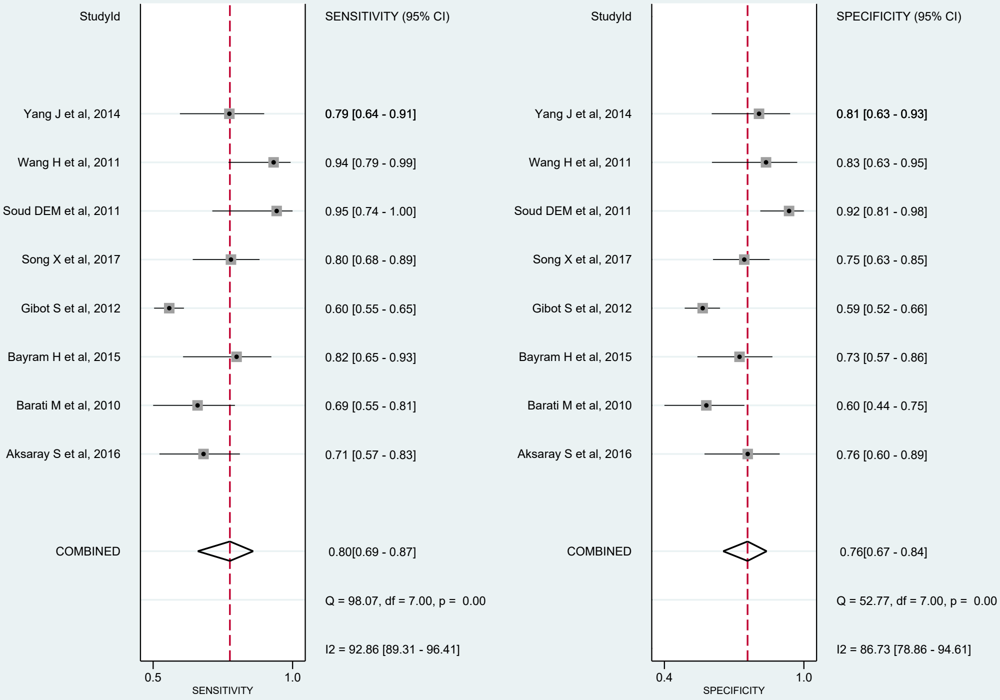

StudyId
SENSITIVITY (95% CI)
Yang J et al, 2014
0.79 [0.64 - 0.91]
Wang H et al, 2011
0.94 [0.79 - 0.99]
Soud DEM et al, 2011
0.95 [0.74 - 1.00]
Song X et al, 2017
0.80 [0.68 - 0.89]
Gibot S et al, 2012
0.60 [0.55 - 0.65]
Bayram H et al, 2015
0.82 [0.65 - 0.93]
Barati M et al, 2010
0.69 [0.55 - 0.81]
Aksaray S et al, 2016
0.71 [0.57 - 0.83]
COMBINED
0.80[0.69 - 0.87]
Q = 98.07, df = 7.00, p = 0.00
I2 = 92.86 [89.31 - 96.41]
0.5
1.0
SENSITIVITY
StudyId
SPECIFICITY (95% CI)
Yang J et al, 2014
0.81 [0.63 - 0.93]
Wang H et al, 2011
0.83 [0.63 - 0.95]
Soud DEM et al, 2011
0.92 [0.81 - 0.98]
Song X et al, 2017
0.75 [0.63 - 0.85]
Gibot S et al, 2012
0.59 [0.52 - 0.66]
Bayram H et al, 2015
0.73 [0.57 - 0.86]
Barati M et al, 2010
0.60 [0.44 - 0.75]
Aksaray S et al, 2016
0.76 [0.60 - 0.89]
COMBINED
0.76[0.67 - 0.84]
Q = 52.77, df = 7.00, p = 0.00
I2 = 86.73 [78.86 - 94.61]
0.4
1.0
SPECIFICITY

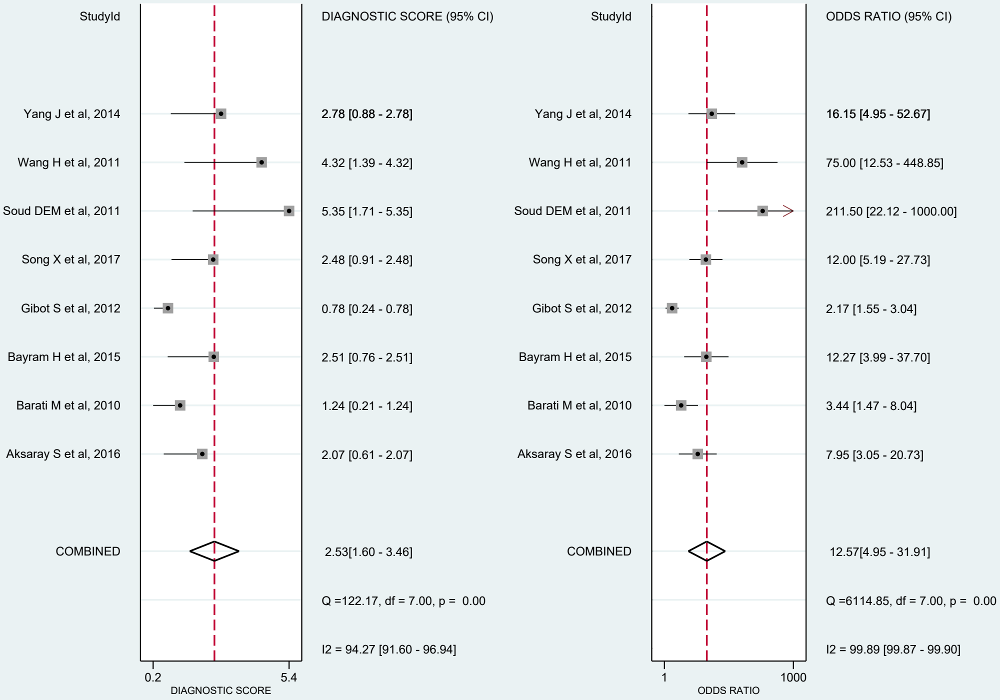
StudyId
DIAGNOSTIC SCORE (95% CI)
Yang J et al, 2014
2.78 [0.88 - 2.78]
Wang H et al, 2011
4.32 [1.39 - 4.32]
Soud DEM et al, 2011
5.35 [1.71 - 5.35]
Song X et al, 2017
2.48 [0.91 - 2.48]
Gibot S et al, 2012
0.78 [0.24 - 0.78]
Bayram H et al, 2015
2.51 [0.76 - 2.51]
Barati M et al, 2010
1.24 [0.21 - 1.24]
Aksaray S et al, 2016
2.07 [0.61 - 2.07]
COMBINED
2.53[1.60 - 3.46]
Q =122.17, df = 7.00, p = 0.00
I2 = 94.27 [91.60 - 96.94]
0.2
5.4
DIAGNOSTIC SCORE
StudyId
ODDS RATIO (95% CI)
Yang J et al, 2014
16.15 [4.95 - 52.67]
Wang H et al, 2011
75.00 [12.53 - 448.85]
Soud DEM et al, 2011
211.50 [22.12 - 1000.00]
Song X et al, 2017
12.00 [5.19 - 27.73]
Gibot S et al, 2012
2.17 [1.55 - 3.04]
Bayram H et al, 2015
12.27 [3.99 - 37.70]
Barati M et al, 2010
3.44 [1.47 - 8.04]
Aksaray S et al, 2016
7.95 [3.05 - 20.73]
COMBINED
12.57[4.95 - 31.91]
Q =6114.85, df = 7.00, p = 0.00
I2 = 99.89 [99.87 - 99.90]
1
1000
ODDS RATIO

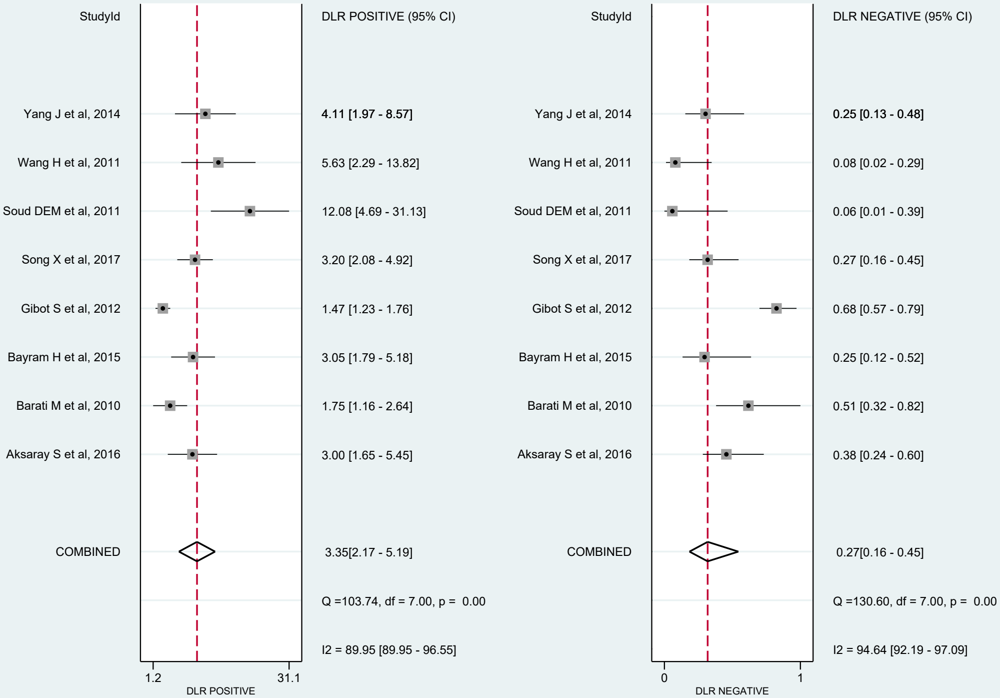
StudyId
DLR POSITIVE (95% CI)
Yang J et al, 2014
4.11 [1.97 - 8.57]
Wang H et al, 2011
5.63 [2.29 - 13.82]
Soud DEM et al, 2011
12.08 [4.69 - 31.13]
Song X et al, 2017
3.20 [2.08 - 4.92]
Gibot S et al, 2012
1.47 [1.23 - 1.76]
Bayram H et al, 2015
3.05 [1.79 - 5.18]
Barati M et al, 2010
1.75 [1.16 - 2.64]
Aksaray S et al, 2016
3.00 [1.65 - 5.45]
COMBINED
3.35[2.17 - 5.19]
Q =103.74, df = 7.00, p = 0.00
I2 = 89.95 [89.95 - 96.55]
1.2
31.1
DLR POSITIVE
StudyId
DLR NEGATIVE (95% CI)
Yang J et al, 2014
0.25 [0.13 - 0.48]
Wang H et al, 2011
0.08 [0.02 - 0.29]
Soud DEM et al, 2011
0.06 [0.01 - 0.39]
Song X et al, 2017
0.27 [0.16 - 0.45]
Gibot S et al, 2012
0.68 [0.57 - 0.79]
Bayram H et al, 2015
0.25 [0.12 - 0.52]
Barati M et al, 2010
0.51 [0.32 - 0.82]
Aksaray S et al, 2016
0.38 [0.24 - 0.60]
COMBINED
0.27[0.16 - 0.45]
Q =130.60, df = 7.00, p = 0.00
I2 = 94.64 [92.19 - 97.09]
0
1
DLR NEGATIVE

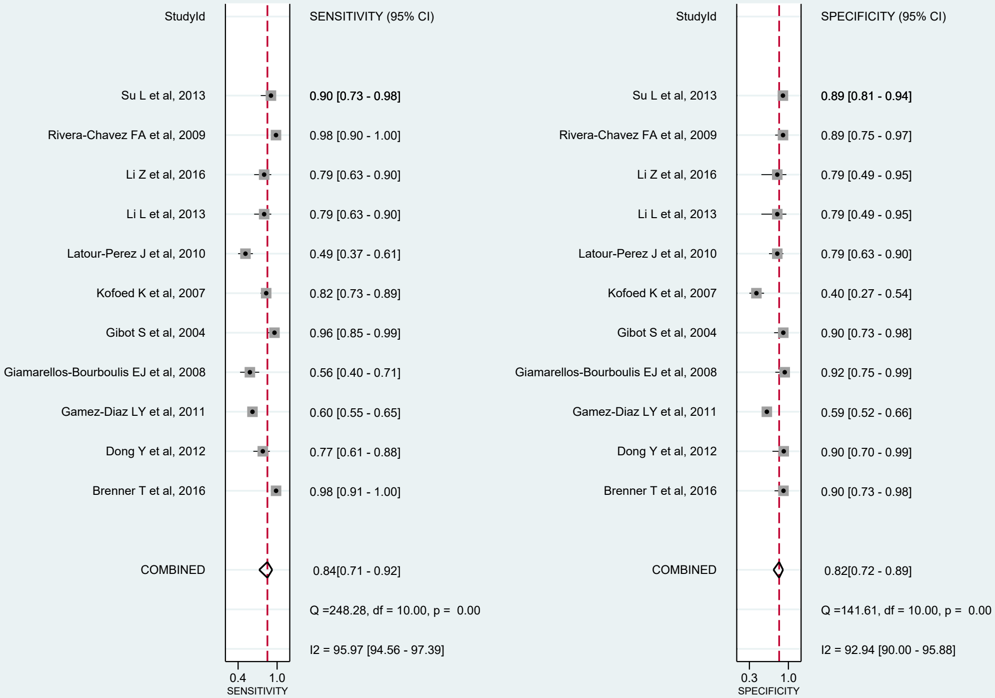
StudyId
SENSITIVITY (95% CI)
Su L et al, 2013
0.90 [0.73 - 0.98]
Rivera-Chavez FA et al, 2009
0.98 [0.90 - 1.00]
Li Z et al, 2016
0.79 [0.63 - 0.90]
Li L et al, 2013
0.79 [0.63 - 0.90]
Latour-Perez J et al, 2010
0.49 [0.37 - 0.61]
Kofoed K et al, 2007
0.82 [0.73 - 0.89]
Gibot S et al, 2004
0.96 [0.85 - 0.99]
Giamarellos-Bourboulis EJ et al, 2008
0.56 [0.40 - 0.71]
Gamez-Diaz LY et al, 2011
0.60 [0.55 - 0.65]
Dong Y et al, 2012
0.77 [0.61 - 0.88]
Brenner T et al, 2016
0.98 [0.91 - 1.00]
COMBINED
0.84[0.71 - 0.92]
Q =248.28, df = 10.00, p = 0.00
I2 = 95.97 [94.56 - 97.39]
0.4
1.0
SENSITIVITY
StudyId
SPECIFICITY (95% CI)
Su L et al, 2013
0.89 [0.81 - 0.94]
Rivera-Chavez FA et al, 2009
0.89 [0.75 - 0.97]
Li Z et al, 2016
0.79 [0.49 - 0.95]
Li L et al, 2013
0.79 [0.49 - 0.95]
Latour-Perez J et al, 2010
0.79 [0.63 - 0.90]
Kofoed K et al, 2007
0.40 [0.27 - 0.54]
Gibot S et al, 2004
0.90 [0.73 - 0.98]
Giamarellos-Bourboulis EJ et al, 2008
0.92 [0.75 - 0.99]
Gamez-Diaz LY et al, 2011
0.59 [0.52 - 0.66]
Dong Y et al, 2012
0.90 [0.70 - 0.99]
Brenner T et al, 2016
0.90 [0.73 - 0.98]
COMBINED
0.82[0.72 - 0.89]
Q =141.61, df = 10.00, p = 0.00
I2 = 92.94 [90.00 - 95.88]
0.3
1.0
SPECIFICITY

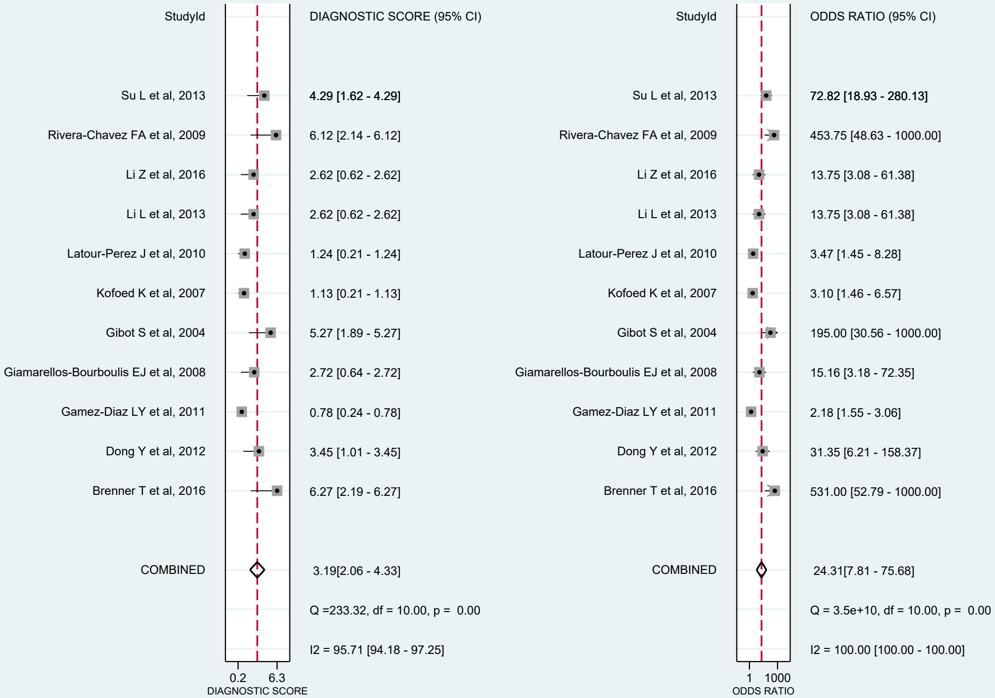
StudyId
DIAGNOSTIC SCORE (95% CI)
Su L et al, 2013
4.29 [1.62 - 4.29]
Rivera-Chavez FA et al, 2009
6.12 [2.14 - 6.12]
Li Z et al, 2016
2.62 [0.62 - 2.62]
Li L et al, 2013
2.62 [0.62 - 2.62]
Latour-Perez J et al, 2010
1.24 [0.21 - 1.24]
Kofoed K et al, 2007
1.13 [0.21 - 1.13]
Gibot S et al, 2004
5.27 [1.89 - 5.27]
Giamarellos-Bourboulis EJ et al, 2008
2.72 [0.64 - 2.72]
Gamez-Diaz LY et al, 2011
0.78 [0.24 - 0.78]
Dong Y et al, 2012
3.45 [1.01 - 3.45]
Brenner T et al, 2016
6.27 [2.19 - 6.27]
COMBINED
3.19[2.06 - 4.33]
Q =233.32, df = 10.00, p = 0.00
I2 = 95.71 [94.18 - 97.25]
0.2
6.3
DIAGNOSTIC SCORE
StudyId
ODDS RATIO (95% CI)
Su L et al, 2013
72.82 [18.93 - 280.13]
Rivera-Chavez FA et al, 2009
453.75 [48.63 - 1000.00]
Li Z et al, 2016
13.75 [3.08 - 61.38]
Li L et al, 2013
13.75 [3.08 - 61.38]
Latour-Perez J et al, 2010
3.47 [1.45 - 8.28]
Kofoed K et al, 2007
3.10 [1.46 - 6.57]
Gibot S et al, 2004
195.00 [30.56 - 1000.00]
Giamarellos-Bourboulis EJ et al, 2008
15.16 [3.18 - 72.35]
Gamez-Diaz LY et al, 2011
2.18 [1.55 - 3.06]
Dong Y et al, 2012
31.35 [6.21 - 158.37]
Brenner T et al, 2016
531.00 [52.79 - 1000.00]
COMBINED
24.31[7.81 - 75.68]
Q = 3.5e+10, df = 10.00, p = 0.00
I2 = 100.00 [100.00 - 100.00]
1
1000
ODDS RATIO

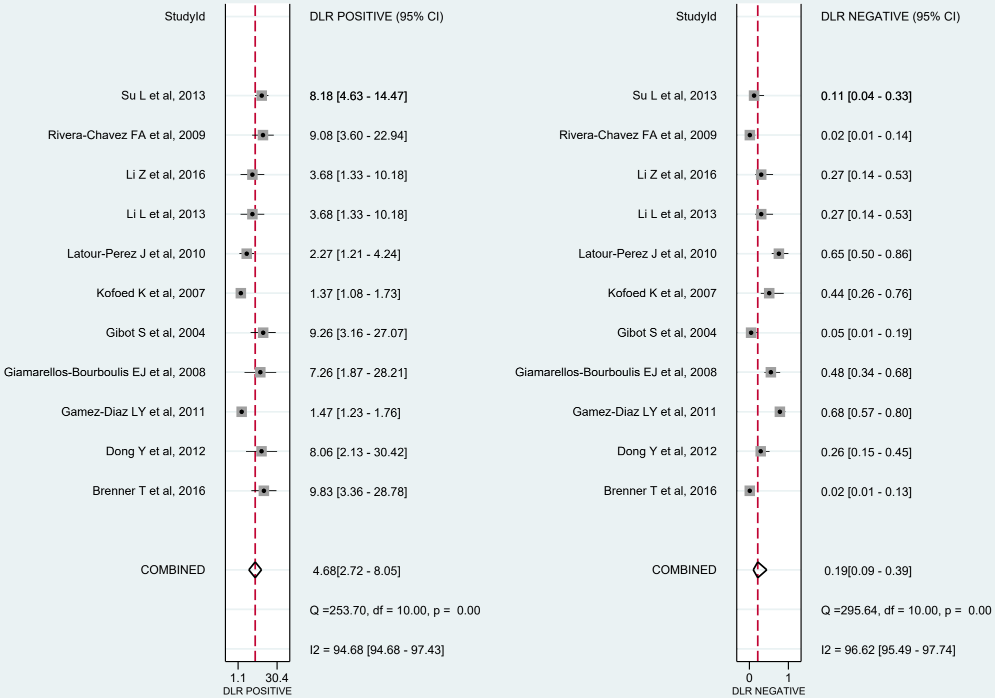
StudyId
DLR POSITIVE (95% CI)
Su L et al, 2013
8.18 [4.63 - 14.47]
Rivera-Chavez FA et al, 2009
9.08 [3.60 - 22.94]
Li Z et al, 2016
3.68 [1.33 - 10.18]
Li L et al, 2013
3.68 [1.33 - 10.18]
Latour-Perez J et al, 2010
2.27 [1.21 - 4.24]
Kofoed K et al, 2007
1.37 [1.08 - 1.73]
Gibot S et al, 2004
9.26 [3.16 - 27.07]
Giamarellos-Bourboulis EJ et al, 2008
7.26 [1.87 - 28.21]
Gamez-Diaz LY et al, 2011
1.47 [1.23 - 1.76]
Dong Y et al, 2012
8.06 [2.13 - 30.42]
Brenner T et al, 2016
9.83 [3.36 - 28.78]
COMBINED
4.68[2.72 - 8.05]
Q =253.70, df = 10.00, p = 0.00
I2 = 94.68 [94.68 - 97.43]
1.1
30.4
DLR POSITIVE
StudyId
DLR NEGATIVE (95% CI)
Su L et al, 2013
0.11 [0.04 - 0.33]
Rivera-Chavez FA et al, 2009
0.02 [0.01 - 0.14]
Li Z et al, 2016
0.27 [0.14 - 0.53]
Li L et al, 2013
0.27 [0.14 - 0.53]
Latour-Perez J et al, 2010
0.65 [0.50 - 0.86]
Kofoed K et al, 2007
0.44 [0.26 - 0.76]
Gibot S et al, 2004
0.05 [0.01 - 0.19]
Giamarellos-Bourboulis EJ et al, 2008
0.48 [0.34 - 0.68]
Gamez-Diaz LY et al, 2011
0.68 [0.57 - 0.80]
Dong Y et al, 2012
0.26 [0.15 - 0.45]
Brenner T et al, 2016
0.02 [0.01 - 0.13]
COMBINED
0.19[0.09 - 0.39]
Q =295.64, df = 10.00, p = 0.00
I2 = 96.62 [95.49 - 97.74]
0
1
DLR NEGATIVE

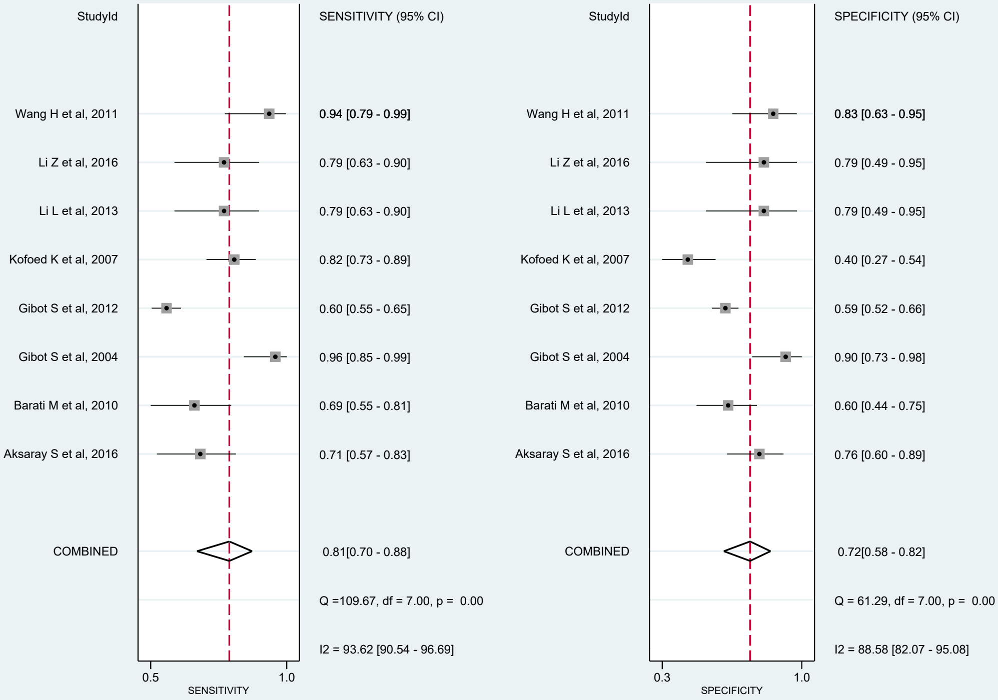
StudyId
SENSITIVITY (95% CI)
Wang H et al, 2011
0.94 [0.79 - 0.99]
Li Z et al, 2016
0.79 [0.63 - 0.90]
Li L et al, 2013
0.79 [0.63 - 0.90]
Kofoed K et al, 2007
0.82 [0.73 - 0.89]
Gibot S et al, 2012
0.60 [0.55 - 0.65]
Gibot S et al, 2004
0.96 [0.85 - 0.99]
Barati M et al, 2010
0.69 [0.55 - 0.81]
Aksaray S et al, 2016
0.71 [0.57 - 0.83]
COMBINED
0.81[0.70 - 0.88]
Q =109.67, df = 7.00, p = 0.00
I2 = 93.62 [90.54 - 96.69]
0.5
1.0
SENSITIVITY
StudyId
SPECIFICITY (95% CI)
Wang H et al, 2011
0.83 [0.63 - 0.95]
Li Z et al, 2016
0.79 [0.49 - 0.95]
Li L et al, 2013
0.79 [0.49 - 0.95]
Kofoed K et al, 2007
0.40 [0.27 - 0.54]
Gibot S et al, 2012
0.59 [0.52 - 0.66]
Gibot S et al, 2004
0.90 [0.73 - 0.98]
Barati M et al, 2010
0.60 [0.44 - 0.75]
Aksaray S et al, 2016
0.76 [0.60 - 0.89]
COMBINED
0.72[0.58 - 0.82]
Q = 61.29, df = 7.00, p = 0.00
I2 = 88.58 [82.07 - 95.08]
0.3
1.0
SPECIFICITY

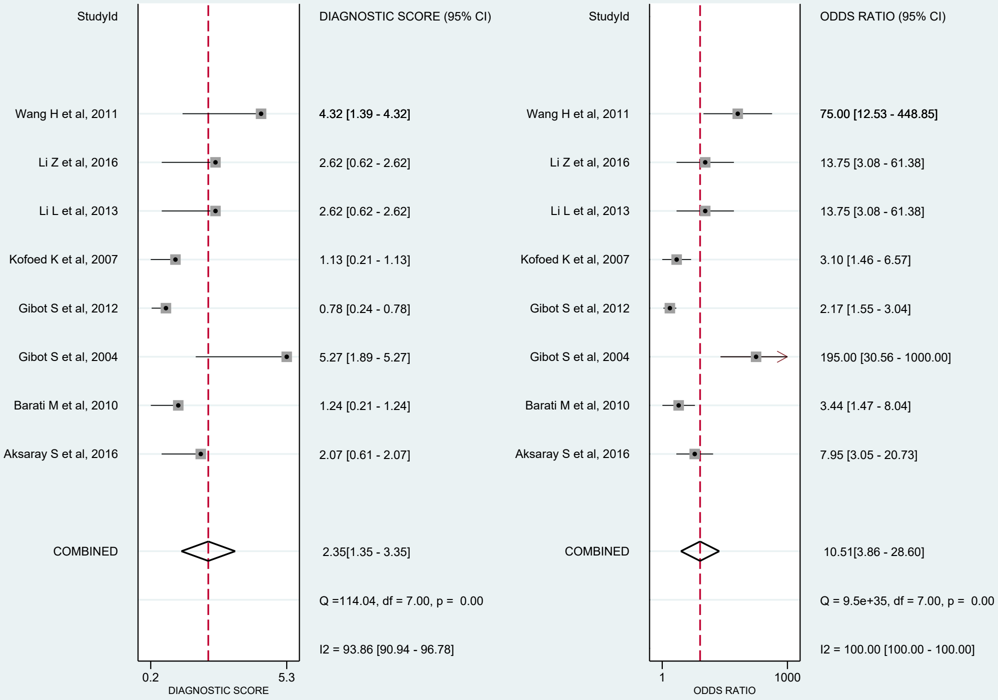

StudyId
DIAGNOSTIC SCORE (95% CI)
Wang H et al, 2011
4.32 [1.39 - 4.32]
Li Z et al, 2016
2.62 [0.62 - 2.62]
Li L et al, 2013
2.62 [0.62 - 2.62]
Kofoed K et al, 2007
1.13 [0.21 - 1.13]
Gibot S et al, 2012
0.78 [0.24 - 0.78]
Gibot S et al, 2004
5.27 [1.89 - 5.27]
Barati M et al, 2010
1.24 [0.21 - 1.24]
Aksaray S et al, 2016
2.07 [0.61 - 2.07]
COMBINED
2.35[1.35 - 3.35]
Q =114.04, df = 7.00, p = 0.00
I2 = 93.86 [90.94 - 96.78]
0.2
5.3
DIAGNOSTIC SCORE
StudyId
ODDS RATIO (95% CI)
Wang H et al, 2011
75.00 [12.53 - 448.85]
Li Z et al, 2016
13.75 [3.08 - 61.38]
Li L et al, 2013
13.75 [3.08 - 61.38]
Kofoed K et al, 2007
3.10 [1.46 - 6.57]
Gibot S et al, 2012
2.17 [1.55 - 3.04]
Gibot S et al, 2004
195.00 [30.56 - 1000.00]
Barati M et al, 2010
3.44 [1.47 - 8.04]
Aksaray S et al, 2016
7.95 [3.05 - 20.73]
COMBINED
10.51[3.86 - 28.60]
Q = 9.5e+35, df = 7.00, p = 0.00
I2 = 100.00 [100.00 - 100.00]
1
1000
ODDS RATIO

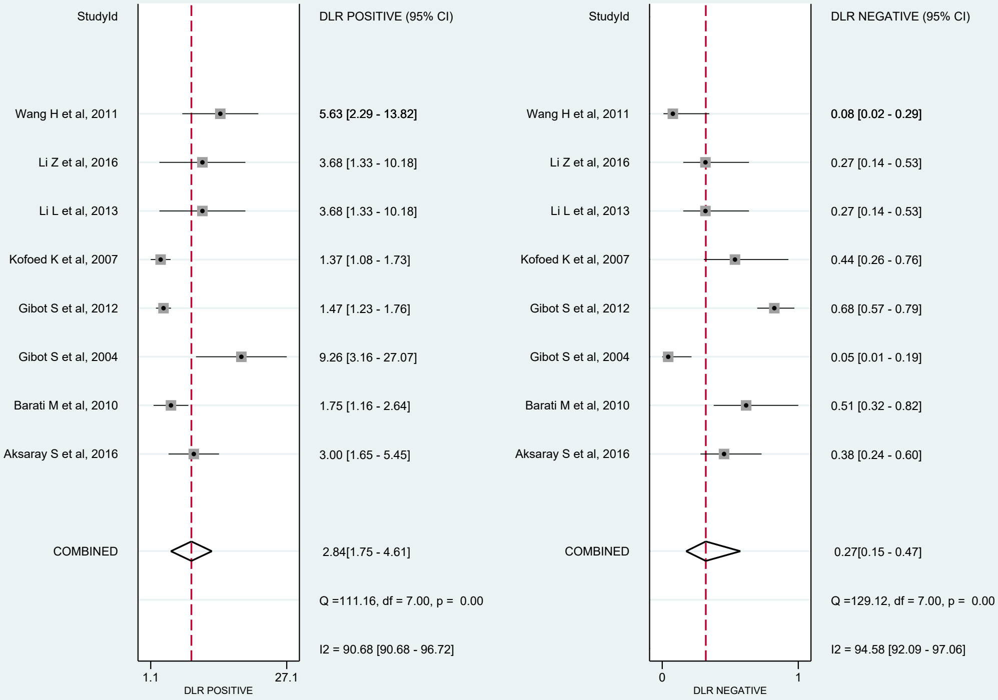
StudyId
DLR POSITIVE (95% CI)
Wang H et al, 2011
5.63 [2.29 - 13.82]
Li Z et al, 2016
3.68 [1.33 - 10.18]
Li L et al, 2013
3.68 [1.33 - 10.18]
Kofoed K et al, 2007
1.37 [1.08 - 1.73]
Gibot S et al, 2012
1.47 [1.23 - 1.76]
Gibot S et al, 2004
9.26 [3.16 - 27.07]
Barati M et al, 2010
1.75 [1.16 - 2.64]
Aksaray S et al, 2016
3.00 [1.65 - 5.45]
COMBINED
2.84[1.75 - 4.61]
Q =111.16, df = 7.00, p = 0.00
I2 = 90.68 [90.68 - 96.72]
1.1
27.1
DLR POSITIVE
StudyId
DLR NEGATIVE (95% CI)
Wang H et al, 2011
0.08 [0.02 - 0.29]
Li Z et al, 2016
0.27 [0.14 - 0.53]
Li L et al, 2013
0.27 [0.14 - 0.53]
Kofoed K et al, 2007
0.44 [0.26 - 0.76]
Gibot S et al, 2012
0.68 [0.57 - 0.79]
Gibot S et al, 2004
0.05 [0.01 - 0.19]
Barati M et al, 2010
0.51 [0.32 - 0.82]
Aksaray S et al, 2016
0.38 [0.24 - 0.60]
COMBINED
0.27[0.15 - 0.47]
Q =129.12, df = 7.00, p = 0.00
I2 = 94.58 [92.09 - 97.06]
0
1
DLR NEGATIVE
